# Supplementary material for: T cell cholesterol efflux suppresses apoptosis and senescence and increases atherosclerosis in middle aged mice
Source: Nat Commun. 2022 Jul 1;13:3799. doi: 10.1038/s41467-022-31135-4 (PMC9249754; doi:10.1038/s41467-022-31135-4)
Supplement: Supplementary file 1 — Supplementary Information [file 41467_2022_31135_MOESM1_ESM.pdf]

SUPPLEMENTARY INFORMATION

T cell cholesterol efflux suppresses apoptosis and senescence and increases atherosclerosis in middle aged mice

Venetia Bazioti[1,2], Anouk M. La Rose[1], Sjors Maassen[3], Frans Bianchi[3], Rinse de Boer[3], Benedek Halmos[1], Deepti Dabral[3], Emma Guilbaud[4], Arthur Flohr-Svendsen[5], Anouk G. Groenen[1], Alejandro Marmolejo-Garza[1], Mirjam H. Koster[1], Niels J. Kloosterhuis[1], Rick Havinga[1], Alle T. Pranger[6], Miriam Langelaar-Makkinje[1], Alain de Bruin[1,7], Bart van de Sluis[1], Alison B. Kohan[8], Laurent Yvan-Charvet[4], Geert van den Bogaart[3], and Marit Westerterp[1,*]

[1] Department of Pediatrics, University Medical Center Groningen, University of Groningen, Groningen, 9713 AV, the Netherlands

[2] Institute for Cardiovascular Prevention (IPEK), Ludwig-Maximilians-Universität, Munich, 80336, Germany

[3] Department of Molecular Immunology and Microbiology, Groningen Biomolecular Sciences and Biotechnology Institute, University of Groningen, Groningen, 9747 AG, the Netherlands

[4] Institut National de la Santé et de la Recherche Médicale (INSERM) U1065, Université Côte d'Azur, Centre Méditerranéen de Médecine Moléculaire (C3M), Atip-Avenir, Fédération Hospitalo-Universitaire (FHU) Oncoage, 06204 Nice, France

[5] European Research Institute for the Biology of Ageing, University Medical Center Groningen, University of Groningen, Groningen, 9713 AV, the Netherlands

[6] Laboratory of Medicine, University Medical Center Groningen, University of Groningen, Groningen, 9713 AV, the Netherlands

[7] Department of Biomolecular Health Sciences, Dutch Molecular Pathology Center, Faculty of Veterinary Medicine, Utrecht University, Utrecht, 3584 CL, the Netherlands

[8] Division of Endocrinology and Metabolism, Department of Medicine, University of Pittsburgh, Pittsburgh, PA 15260, United States

*Correspondence: m.westerterp@umcg.nl

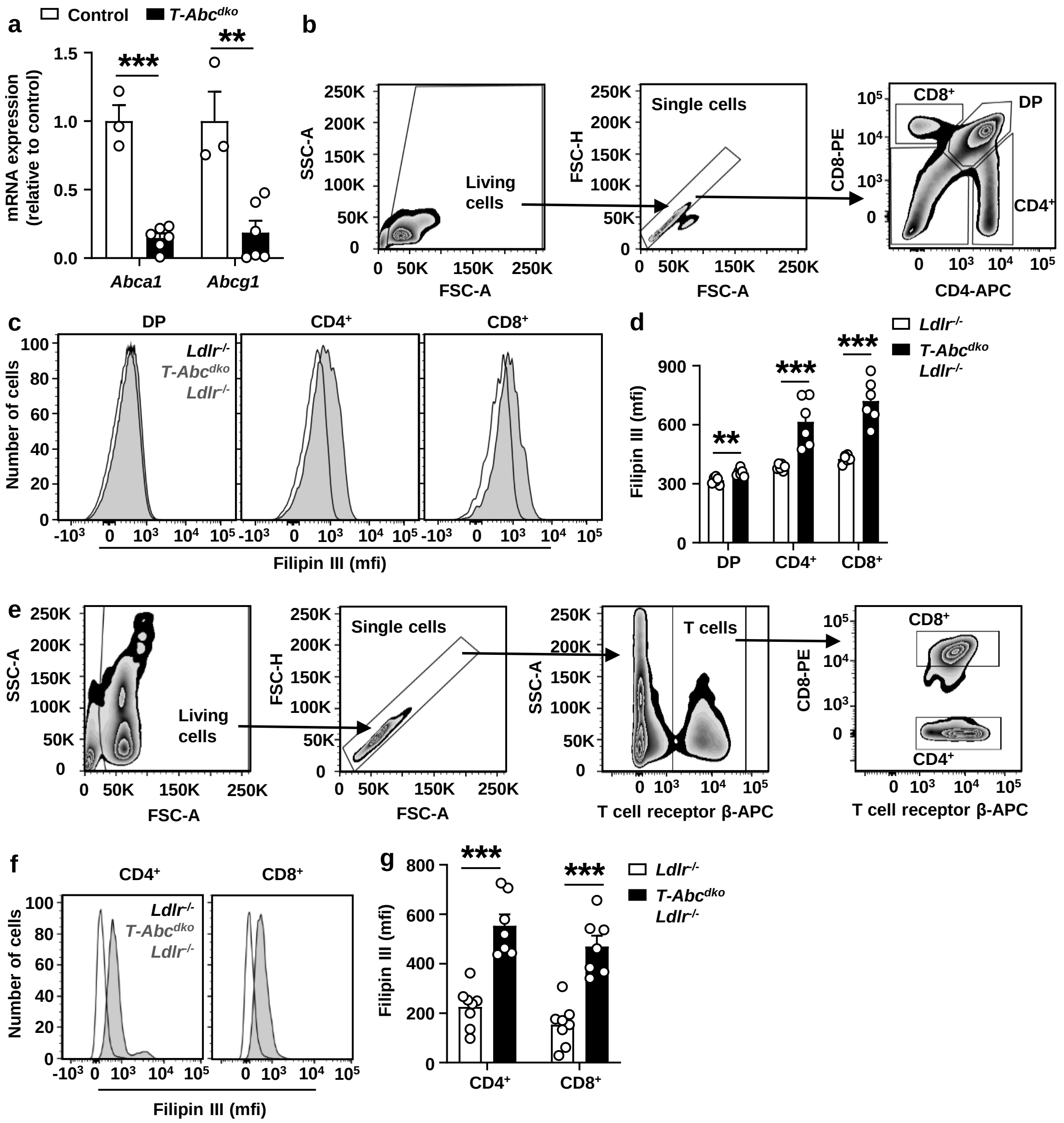

Supplementary Fig. 1 T cell *Abca1/Abcg1* deficiency increases free cholesterol accumulation. Control (*Ldlr*$^{+/+}$), *T-Abc*^{dko}, *Ldlr*$^{-/-}$, and *T-Abc*^{dko}*Ldlr*$^{-/-}$ mice were fed a chow diet. Spleens, thymi, and blood were collected. (**a**) Splenic T cells were isolated and RNA was extracted. *Abca1* (p=0.00003) and *Abcg1* (p=0.00359) mRNA expression were measured by qPCR. n=3 control and n=6 *T-Abc*^{dko} mice. (**b-d**) Filipin staining on thymocytes. (**b**) Gating strategy, (**c**) representative flow cytometry plots, and (**d**) quantification of filipin on CD4^{+}CD8^{+} double positive (DP) cells (p=0.0034), CD4^{+} (p=0.00107), and CD8^{+} (p=0.00008) single positive (SP) cells. n=6 *Ldlr*$^{-/-}$ and n=6 *T-Abc*^{dko}*Ldlr*$^{-/-}$ mice. (**e-g**) Filipin staining on blood T cells. (**e**) Gating strategy, (**f**) representative flow cytometry plots, and (**g**) quantification of filipin staining on CD4^{+} (p=0.00003) and CD8^{+} (p=0.00003) T cells. n=8 *Ldlr*$^{-/-}$ and n=8 *T-Abc*^{dko}*Ldlr*$^{-/-}$ mice. For all panels, error bars represent SEM, and biologically independent samples were included. p value was determined by unpaired two-tailed Student's t-test. **p<0.01, ***p<0.001. Source data are provided as a Source Data file.

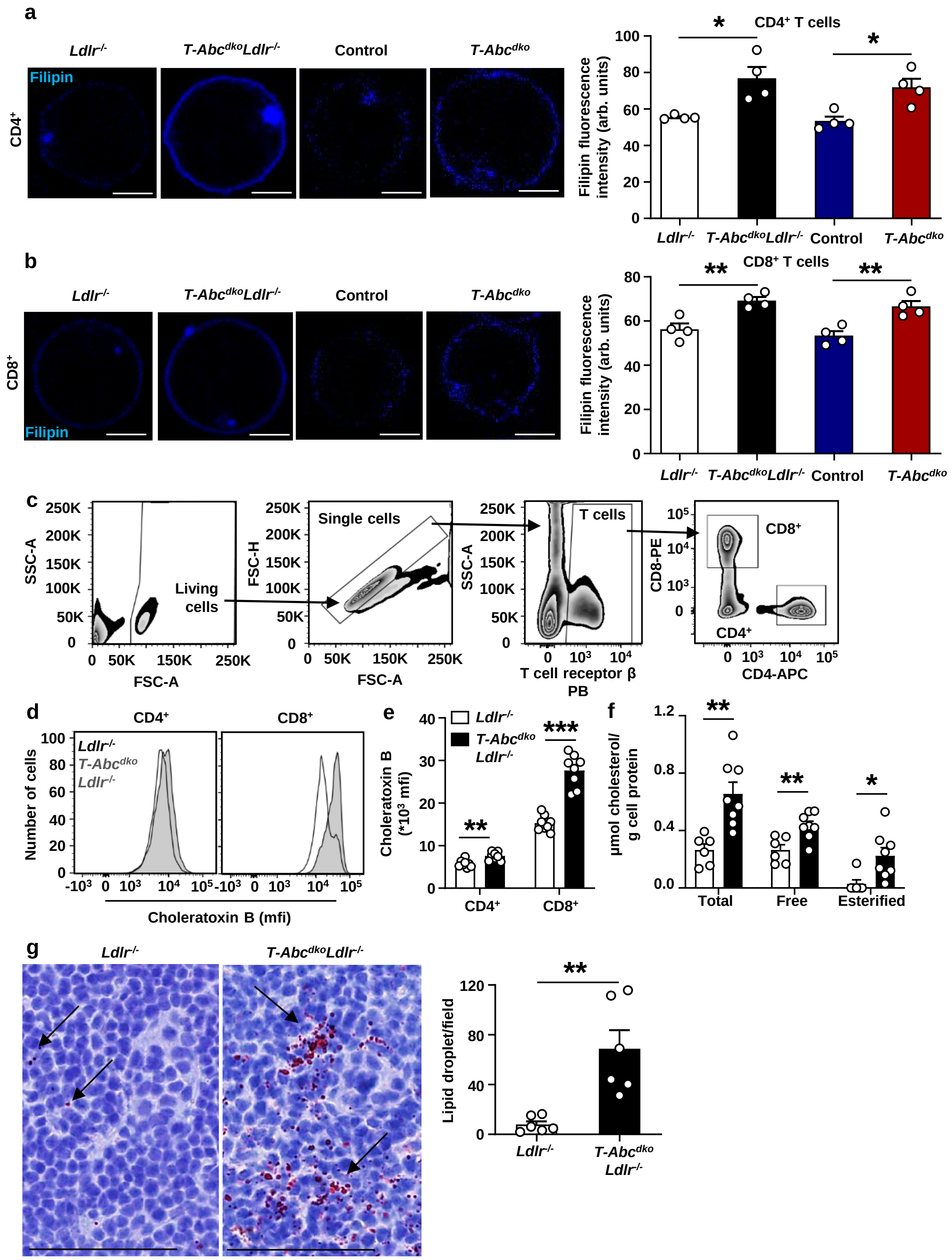

Supplementary Fig. 2 T cell *Abca1/Abcg1* deficiency increases cholesterol accumulation independent of *Ldlr* expression. Control (*Ldlr*$^{+/+}$), *T-Abc*^{dko}, *Ldlr*$^{-/-}$, and *T-Abc*^{dko}*Ldlr*$^{-/-}$ mice were fed a chow diet. Spleens, blood, and para-aortic lymph nodes (LNs) were collected. (**a-b**) Splenic CD4^{+} and CD8^{+} T cells were isolated, fixed, stained with filipin, and analyzed by confocal microscopy. (**a**) Representative pictures (left) and quantification (right) of filipin fluorescence

intensity for CD4$^+$ *Ldlr*$^{-/-}$ and *T-Abc*^{dko}*Ldlr*$^{-/-}$ T cells (*p*=0.0133), and for control and *T-Abc*^{dko} T cells (*p*=0.0127). Per mouse, 8-12 CD4$^+$ T cells were analyzed. n=4 *Ldlr*$^{-/-}$, n=4 *T-Abc*^{dko}*Ldlr*$^{-/-}$, n=4 control, and n=4 *T-Abc*^{dko} mice. (**b**) Representative pictures (left) and quantification (right) of filipin fluorescence intensity for CD8$^+$ *Ldlr*$^{-/-}$ and *T-Abc*^{dko}*Ldlr*$^{-/-}$ T cells (*p*=0.0053), and for control and *T-Abc*^{dko} T cells (*p*=0.0062). Per mouse, 7-8 CD8$^+$ T cells were analyzed. n=4 *Ldlr*$^{-/-}$, n=4 *T-Abc*^{dko}*Ldlr*$^{-/-}$, n=4 control, and n=4 *T-Abc*^{dko} mice. (**a-b**) Scale bar represents 2 μm. (**c-e**) Choleratoxin B staining on blood T cells. (**c**) Gating strategy, (**d**) representative flow cytometry plots, and (**e**) quantification of choleratoxin B on CD4$^+$ (*p*=0.0014) and CD8$^+$ (*p*=0.000001) T cells. n=8 *Ldlr*$^{-/-}$ and n=8 *T-Abc*^{dko}*Ldlr*$^{-/-}$ mice. (**f**) Splenic T cells were isolated and total (*p*=0.002) and free cholesterol (*p*=0.005) were measured by Gas Chromatography – Mass Spectrometry (GC-MS). Esterified cholesterol (*p*=0.018) equals total cholesterol - free cholesterol. n=6 *Ldlr*$^{-/-}$ and n=8 *T-Abc*^{dko}*Ldlr*$^{-/-}$ mice. (**g**) Para-aortic LNs were embedded in OCT and frozen sections were stained for Oil Red O. Representative pictures (left) and quantification (*p*=0.0027) (right) of lipid droplets depicted by arrows. Scale bar represents 60 μm. n=6 *Ldlr*$^{-/-}$ and n=6 *T-Abc*^{dko}*Ldlr*$^{-/-}$ mice. For all panels, error bars represent SEM, and biologically independent samples were included. *p* value was determined by unpaired two-tailed Student's t-test. **p*<0.05, ***p*<0.01, ****p*<0.001. Source data are provided as a Source Data file.

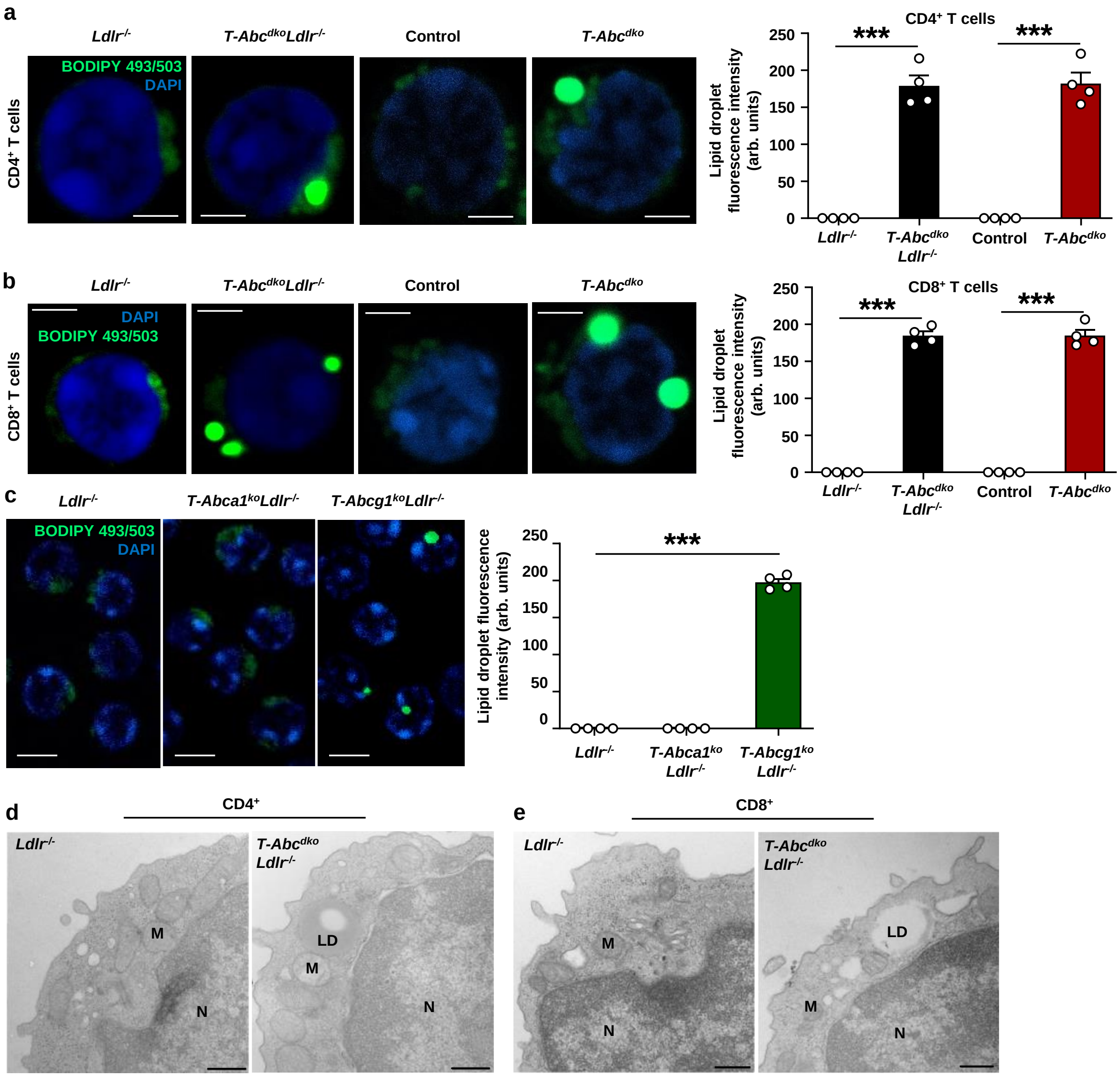

Supplementary Fig. 3 T cell *Abca1/Abcg1* deficiency induces lipid droplet formation. Spleens were collected from control (*Ldlr*$^{+/+}$), *T-Abc*^{dko}, *Ldlr*$^{-/-}$, *T-Abc*^{dko}*Ldlr*$^{-/-}$, *T-Abca1*^{sko}*Ldlr*$^{-/-}$, and *T-Abcg1*^{sko}*Ldlr*$^{-/-}$ mice fed a chow diet. (**a-c**) Splenic CD4^{+} and CD8^{+} T cells, and total T cells were isolated, fixed, permeabilized, stained with BODIPY 493/503 and DAPI, and analyzed by confocal microscopy. (**a**) Representative pictures (left) and quantification (right) of fluorescence intensity of lipid droplets for CD4^{+} *Ldlr*$^{-/-}$ and *T-Abc*^{dko}*Ldlr*$^{-/-}$ T cells (p=0.000013), and for control and *T-Abc*^{dko} T cells (p=0.000016). Per mouse, 6-7 CD4^{+} T cells were analyzed. n=4 *Ldlr*$^{-/-}$, n=4 *T-Abc*^{dko}*Ldlr*$^{-/-}$, n=4 control, and n=4 *T-Abc*^{dko} mice. (**b**) Representative pictures (left) and quantification (right) of fluorescence intensity of lipid droplets for CD8^{+} *Ldlr*$^{-/-}$ and *T-Abc*^{dko}*Ldlr*$^{-/-}$ T cells (p<0.000001), and for control and *T-Abc*^{dko} T cells (p<0.000001). Per mouse, 5-6 CD8^{+} T cells were analyzed. n=4 *Ldlr*$^{-/-}$, n=4 *T-Abc*^{dko}*Ldlr*$^{-/-}$, n=4 control, and n=4 *T-Abc*^{dko} mice. (**c**) Representative pictures (left) and quantification (right) of fluorescence intensity of lipid droplets for *Ldlr*$^{-/-}$, *T-Abca1*^{sko}*Ldlr*$^{-/-}$, and *T-Abcg1*^{sko}*Ldlr*$^{-/-}$ (p<0.0001) T cells. Per mouse, 10 T cells were analyzed. n=4 *Ldlr*$^{-/-}$, n=4 *T-Abca1*^{sko}*Ldlr*$^{-/-}$, and n=4 *T-Abcg1*^{sko}*Ldlr*$^{-/-}$ mice. (**a-c**) Scale bar represents (**a-b**) 2 µm or (**c**) 5 µm. (**d-e**) Representative transmission electron microscopy pictures for (**d**) CD4^{+} and (**e**) CD8^{+} T cells. Per cell type per mouse, 50 cell sections (100 nm thickness) were analyzed. n=3 *Ldlr*$^{-/-}$ and n=3 *T-Abc*^{dko}*Ldlr*$^{-/-}$ mice. LD; lipid droplet; M, mitochondria; N,

nucleus. Scale bar represents 500 nm. For all panels, error bars represent SEM, and biologically independent samples were included. *p* value was determined by unpaired two-tailed Student's t-test (**a-b**) or one-way ANOVA with Bonferroni post-test (**c**). ***$p<0.001$. Source data are provided as a Source Data file.

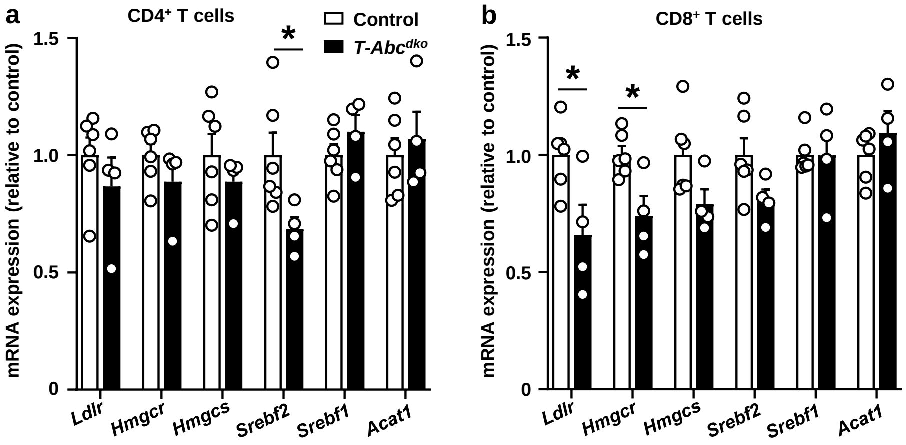

Supplementary Fig. 4 Effects of T cell *Abca1/Abcg1* deficiency on expression of genes involved in lipid metabolism. Spleens were collected from control (*Ldlr*$^{+/+}$) and *T-Abc*^{dko} mice fed a chow diet. CD4$^+$ and CD8$^+$ T cells were isolated and RNA was extracted. Low-density lipoprotein receptor *(Ldlr)* (*p*=0.026), 3-hydroxy-3-methyl-glutaryl-coenzyme A reductase *(Hmgcr)* (*p*=0.013), HMG-CoA synthase *(Hmgcs),* sterol regulatory element binding transcription factor 2 *(Srebf2)* (*p*=0.038), *Srebf1, and* acetyl-CoA cholesterol acyltransferase 1 (*Acat1)* mRNA expression were measured in (**a**) CD4$^+$ and (**b**) CD8$^+$ T cells by qPCR. n=6 control and n=4 *T-Abc*^{dko} mice. For all panels, error bars represent SEM, and biologically independent samples were included. *p* value was determined by unpaired two-tailed Student's t-test. *$p<0.05$. Source data are provided as a Source Data file.

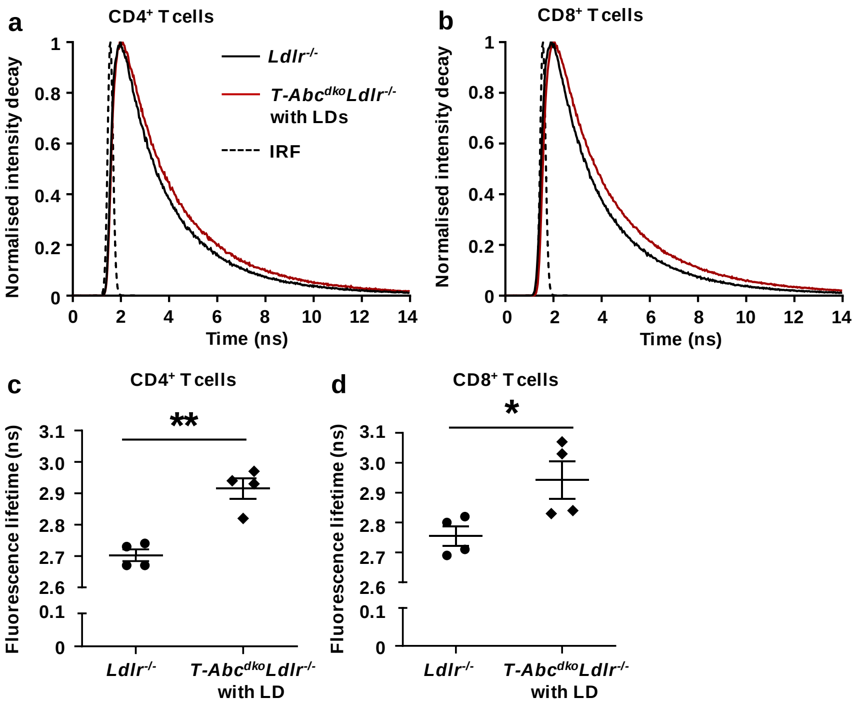

Supplementary Fig. 5 T cell *Abca1/Abcg1* deficiency increases cell stiffness. Spleens were collected from *Ldlr*$^{-/-}$ and *T-Abc*^{dko}*Ldlr*$^{-/-}$ mice fed a chow diet. CD4^{+} and CD8^{+} T cells were isolated, stained with BODIPY C10, and analyzed by Fluorescence-Lifetime Imaging Microscopy (FLIM). (**a-b**) Representative fluorescence lifetime decay curves for pictures for CD4^{+} (**a**) and CD8^{+} (**b**) T cells. LDs, lipid droplets. Dashed lines represent fits with mono-exponential decay functions convoluted with the instrument response function (IRF). (**c-d**) BODIPY C10 fluorescence lifetime (determined by fitting the fluorescence lifetime decay curves with a mono-exponential decay function) for (**c**) CD4^{+} (p=0.0014) and (**d**) CD8^{+} (p=0.037) T cells. Per mouse, 43-76 CD4^{+} and CD8^{+} T cells were analyzed. n=4 *Ldlr*$^{-/-}$ and n=4 *T-Abc*^{dko}*Ldlr*$^{-/-}$ mice. For all panels, error bars represent SEM, and biologically independent samples were included. p value was determined by unpaired two-tailed Student's t-test. *p<0.05, **p<0.01. Source data are provided as a Source Data file.

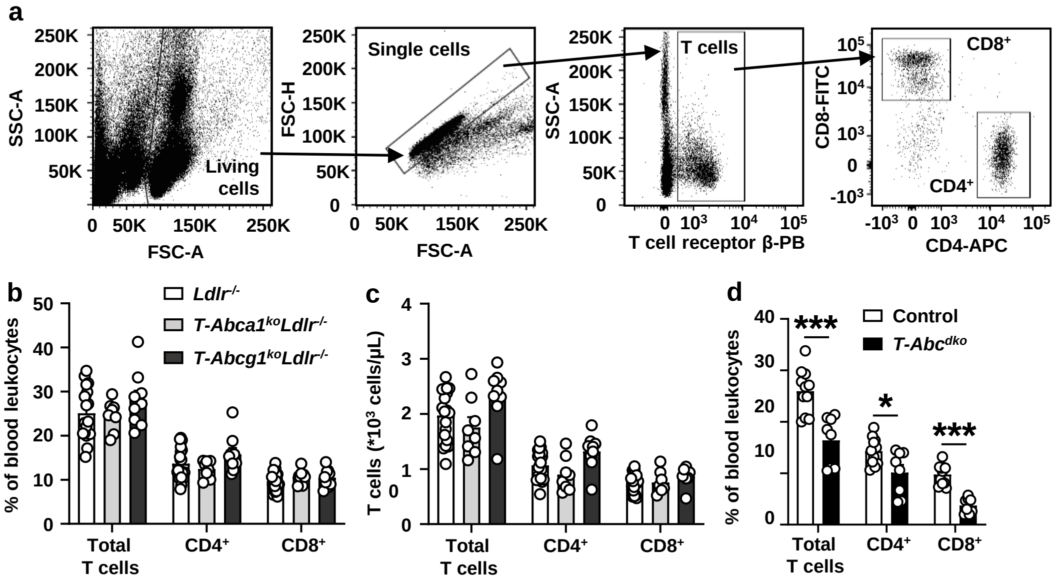

Supplementary Fig. 6 T cell *Abca1/Abcg1* deficiency decreases blood T cells independent of *Ldlr* expression. Blood was collected from *Ldlr*$^{-/-}$, *T-Abca1*^{sko}*Ldlr*$^{-/-}$, *T-Abcg1*^{sko}*Ldlr*$^{-/-}$, control (*Ldlr*$^{+/+}$), *T-Abc*^{dko}, and *T-Abc*^{dko}*Ldlr*$^{-/-}$ mice fed a chow diet. White blood cells (leukocytes) were stained with the indicated antibodies and analyzed by flow cytometry. (**a**) Gating strategy for CD4 and CD8 staining on blood leukocytes. (**b**) Quantification of T cell receptor β (TCRβ)$^+$ (total T cells), CD4$^+$, and CD8$^+$ T cells as a percentage of total blood leukocytes, and (**c**) total T cell, CD4$^+$, and CD8$^+$ T cell concentration in blood after correction for total leukocytes. n=19 *Ldlr*$^{-/-}$, n=8 *T-Abca1*^{sko}*Ldlr*$^{-/-}$, and n=9 *T-Abcg1*^{sko}*Ldlr*$^{-/-}$ mice. (**d**) Total T cells (p=0.0003), CD4$^+$ (p=0.018), and CD8$^+$ (p=0.0000013) T cells as a percentage of total blood leukocytes. n=11 control and n=8 *T-Abc*^{dko} mice. For all panels, error bars represent SEM, and biologically independent samples were included. p value was determined by unpaired two-tailed Student's t-test (**d**) or one-way ANOVA with Bonferroni post-test (**b, c**). *p<0.05, ***p<0.001. Source data are provided as a Source Data file.

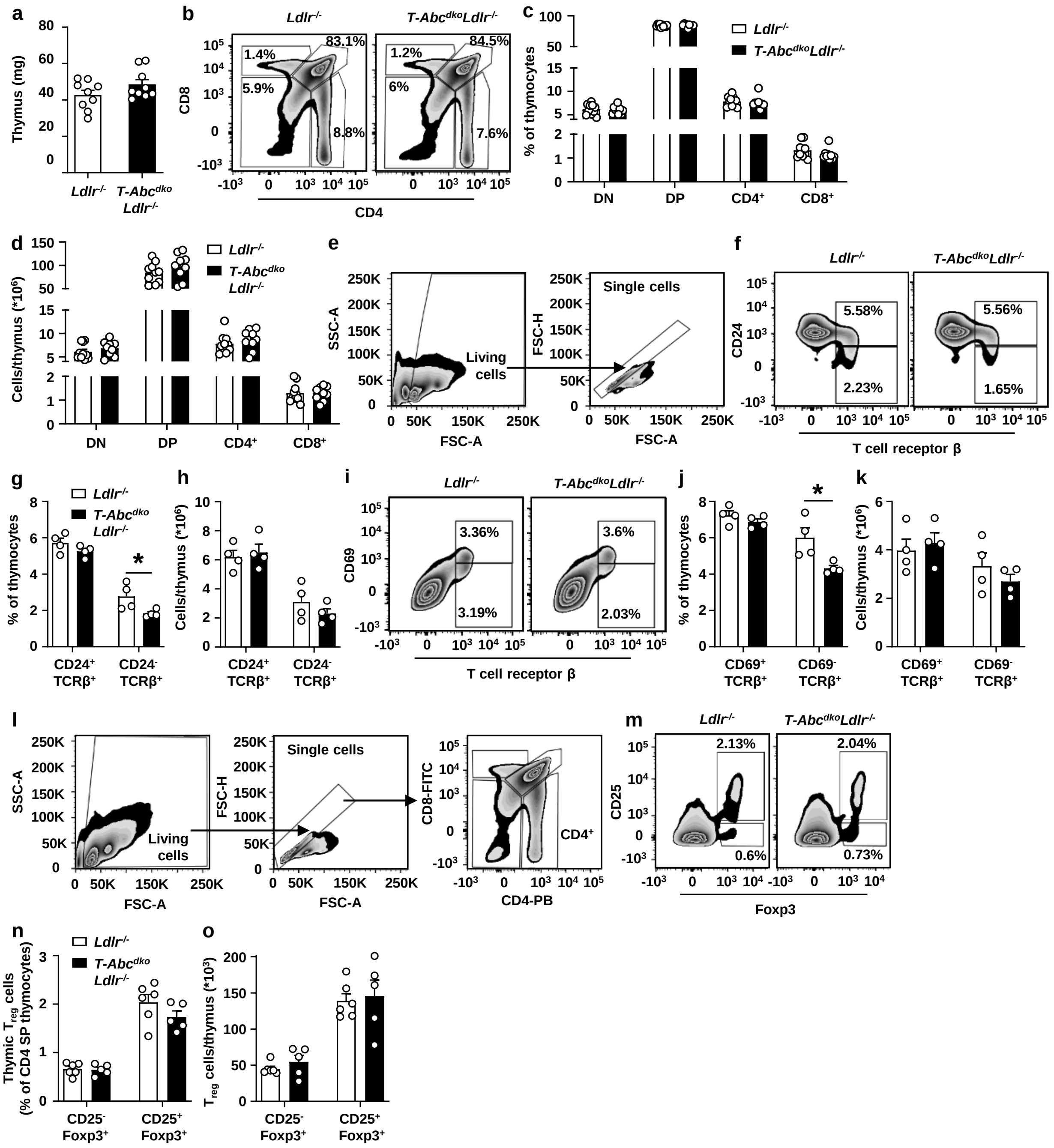

Supplementary Fig. 7 T cell *Abca1/Abcg1* deficiency does not affect thymic T cells. Thymi were collected from *Ldlr*$^{-/-}$ and *T-Abc*^{dko}*Ldlr*$^{-/-}$ mice fed a chow diet. (**a**) Thymic weight. n=9 *Ldlr*$^{-/-}$ and n=9 *T-Abc*^{dko}*Ldlr*$^{-/-}$ mice. (**b-o**) Thymi were mashed, stained with the indicated antibodies, and analyzed by flow cytometry. (**b-d**) CD4 and CD8 staining on thymocytes. (**b**) Representative flow cytometry plots of thymic CD4^{-}CD8^{-} double negative (DN), CD4^{+}CD8^{+} DP, CD4^{+}, and CD8^{+} SP cells gated as in Supplementary Fig. 1b. (**c**) DN, DP, CD4^{+} and CD8^{+} SP cells as a percentage of total thymocytes, and (**d**) expressed as cells/thymus after correction for total thymocyte numbers. n=10 *Ldlr*$^{-/-}$ and n=9 *T-Abc*^{dko}*Ldlr*$^{-/-}$ mice. (**e-k**) Thymocyte positive/negative selection marker stainings. (**e**) Gating strategy and (**f**) representative flow cytometry plots of thymic TCRβ^{+}CD24^{+} and TCRβ^{+}CD24^{-} cells, (**g**) TCRβ^{+}CD24^{+} and TCRβ^{+}CD24^{-} (*p*=0.046) cells as a percentage of

total thymocytes, and (**h**) expressed as cells/thymus. n=4 *Ldlr*$^{-/-}$ and n=4 *T-Abc*^{dko}*Ldlr*$^{-/-}$ mice. (**i**) Representative flow cytometry plots of thymic TCRβ^{+}CD69^{+} and TCRβ^{+}CD69^{-} cells, (**j**) TCRβ^{+}CD69^{+} and TCRβ^{+}CD69^{-} (p=0.026) cells as a percentage of total thymocytes, and (**k**) expressed as cells/thymus. n=4 *Ldlr*$^{-/-}$ and n=4 *T-Abc*^{dko}*Ldlr*$^{-/-}$ mice. (**l-o**) Thymic $T_{regulatory}$ cells (T_{reg}). (**l**) Gating strategy and (**m**) representative flow cytometry plots of thymic CD25^{-}Foxp3^{+} and CD25^{+}Foxp3^{+} T_{reg} cells, (**n**) CD25^{-}Foxp3^{+} and CD25^{+}Foxp3^{+} T_{reg} cells as a percentage of CD4 SP thymocytes, and (**o**) expressed as cells/thymus. n=6 *Ldlr*$^{-/-}$ and n=5 *T-Abc*^{dko}*Ldlr*$^{-/-}$ mice. For all panels, error bars represent SEM, and biologically independent samples were included. p value was determined by unpaired two-tailed Student's t-test. *p<0.05. Source data are provided as a Source Data file.

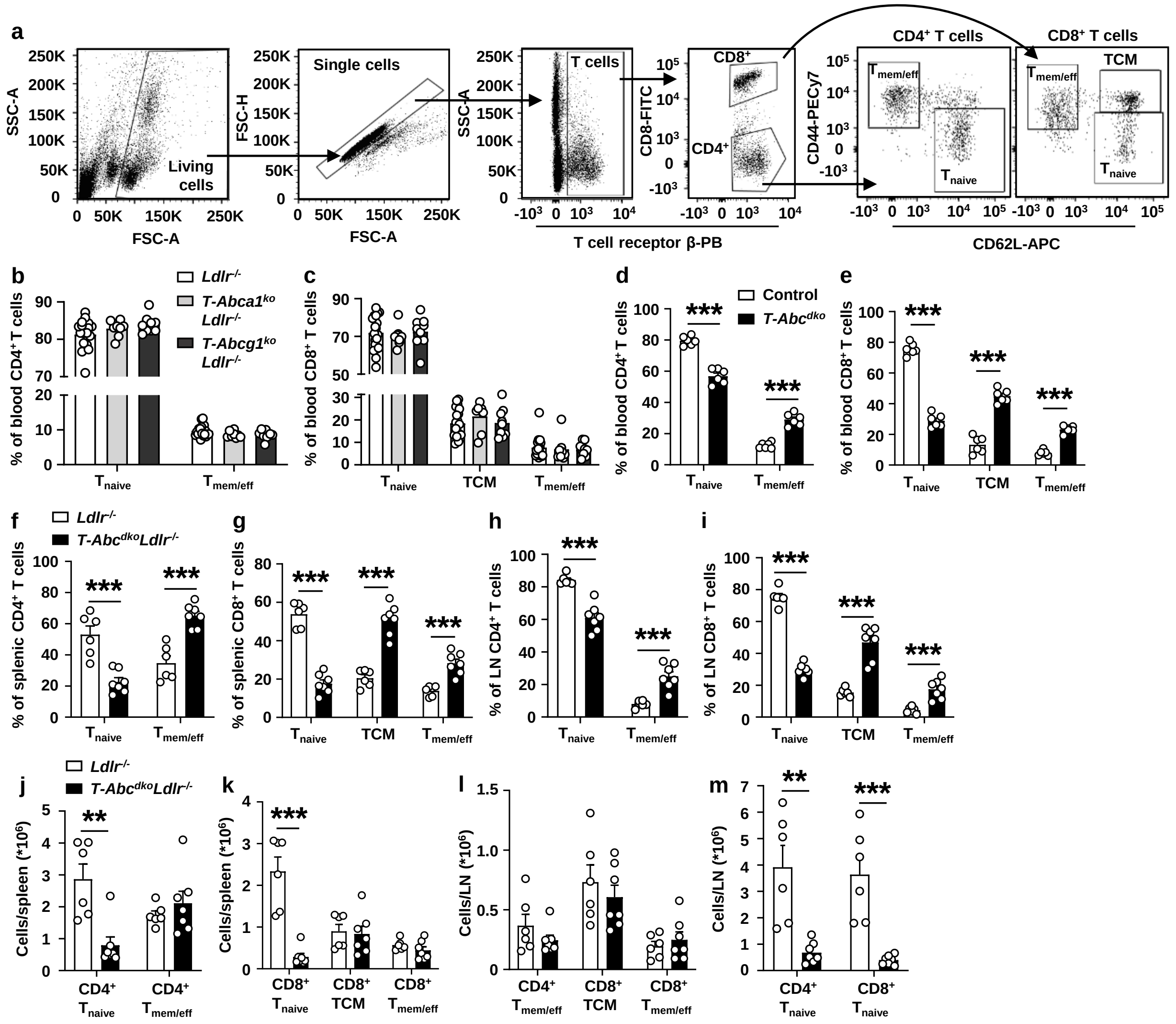

Supplementary Fig. 8 T cell *Abca1/Abcg1* deficiency increases T cell activation. *Ldlr*$^{-/-}$, *T-Abca1*^{sko}*Ldlr*$^{-/-}$, *T-Abcg1*^{sko}*Ldlr*$^{-/-}$, control (*Ldlr*$^{+/+}$), *T-Abc*^{dko}, and *T-Abc*^{dko}*Ldlr*$^{-/-}$ mice were fed a chow diet. Blood, spleens, and para-aortic LNs were collected. Tissues were mashed, stained with the indicated antibodies, and analyzed by flow cytometry. (**a**) Gating strategy for T_{naive} ($CD44^-$ $CD62L^+$), $T_{memory/effector}$ ($T_{mem/eff}$; $CD44^+CD62L^-$), and $T_{central\ memory}$ (TCM; $CD8^+CD44^+CD62L^+$) cells. (**b-c**) $CD4^+T_{naive}$, $T_{mem/eff}$ (**b**), $CD8^+$ T_{naive}, $T_{mem/eff}$, and TCM (**c**) cells as a percentage of $CD4^+$ (**b**) or $CD8^+$ (**c**) T cells. n=19 *Ldlr*$^{-/-}$, n=8 *T-Abca1*^{sko}*Ldlr*$^{-/-}$, and n=9 *T-Abcg1*^{sko}*Ldlr*$^{-/-}$ mice. (**d-e**) $CD4^+$ T_{naive} (p=0.0000019) and $T_{mem/eff}$ (p=0.0000029) (**d**), and $CD8^+$ T_{naive} (p<0.000001), $T_{mem/eff}$ (p<0.000001), and TCM (p<0.000001) (**e**) cells as a percentage of $CD4^+$ (**d**) or $CD8^+$ (**e**) T cells. n=6 control and n=6 *T-Abc*^{dko} mice. (**f-g**) $CD4^+$ T_{naive} (p=0.0003) and $T_{mem/eff}$ (p=0.0001) cells (**f**) and $CD8^+$ T_{naive} (p<0.000001), $T_{mem/eff}$ (p=0.0000046), and TCM (p=0.00015) cells (**g**) as a percentage of splenic $CD4^+$ (**f**) or $CD8^+$ (**g**) T cells. n=6 *Ldlr*$^{-/-}$ and n=7 *T-Abc*^{dko}*Ldlr*$^{-/-}$ mice. (**h-i**) $CD4^+$ T_{naive} (p=0.00004) and $T_{mem/eff}$ (p=0.00026) cells (**h**) and $CD8^+$ T_{naive} (p<0.000001), $T_{mem/eff}$ (p=0.00002), and TCM (p=0.0003) cells (**i**) as a percentage of para-aortic LN $CD4^+$ (**h**) or $CD8^+$ (**i**) T cells. n=6 *Ldlr*$^{-/-}$ and n=7 *T-Abc*^{dko}*Ldlr*$^{-/-}$ mice. (**j-k**) $CD4^+$ T_{naive} (p=0.002) and $T_{mem/eff}$ cells (**j**) and $CD8^+$ T_{naive} (p=0.00006), $T_{mem/eff}$, and TCM cells (**k**) expressed as cells/spleen after correction for total splenic T cell number. n=6 *Ldlr*$^{-/-}$ and n=7 *T-Abc*^{dko}*Ldlr*$^{-/-}$ mice. (**l-m**) $CD4^+$ and $CD8^+$ $T_{mem/eff}$, and $CD8^+$ TCM cells (**l**) and $CD4^+$ (p=0.0016) and $CD8^+$ (p=0.00038) T_{naive} cells (**m**)

expressed as cells/LN after correction for total para-aortic LN cell number. n=6 *Ldlr*$^{-/-}$ and n=7 *T-Abc*^{dko}*Ldlr*$^{-/-}$ mice. For all panels, error bars represent SEM, and biologically independent samples were included. *p* value was determined by unpaired two-tailed Student's t-test (**d-m**) or one-way ANOVA with Bonferroni post-test (**b-c**). $^{**}p<0.01$, $^{***}p<0.001$. Source data are provided as a Source Data file.

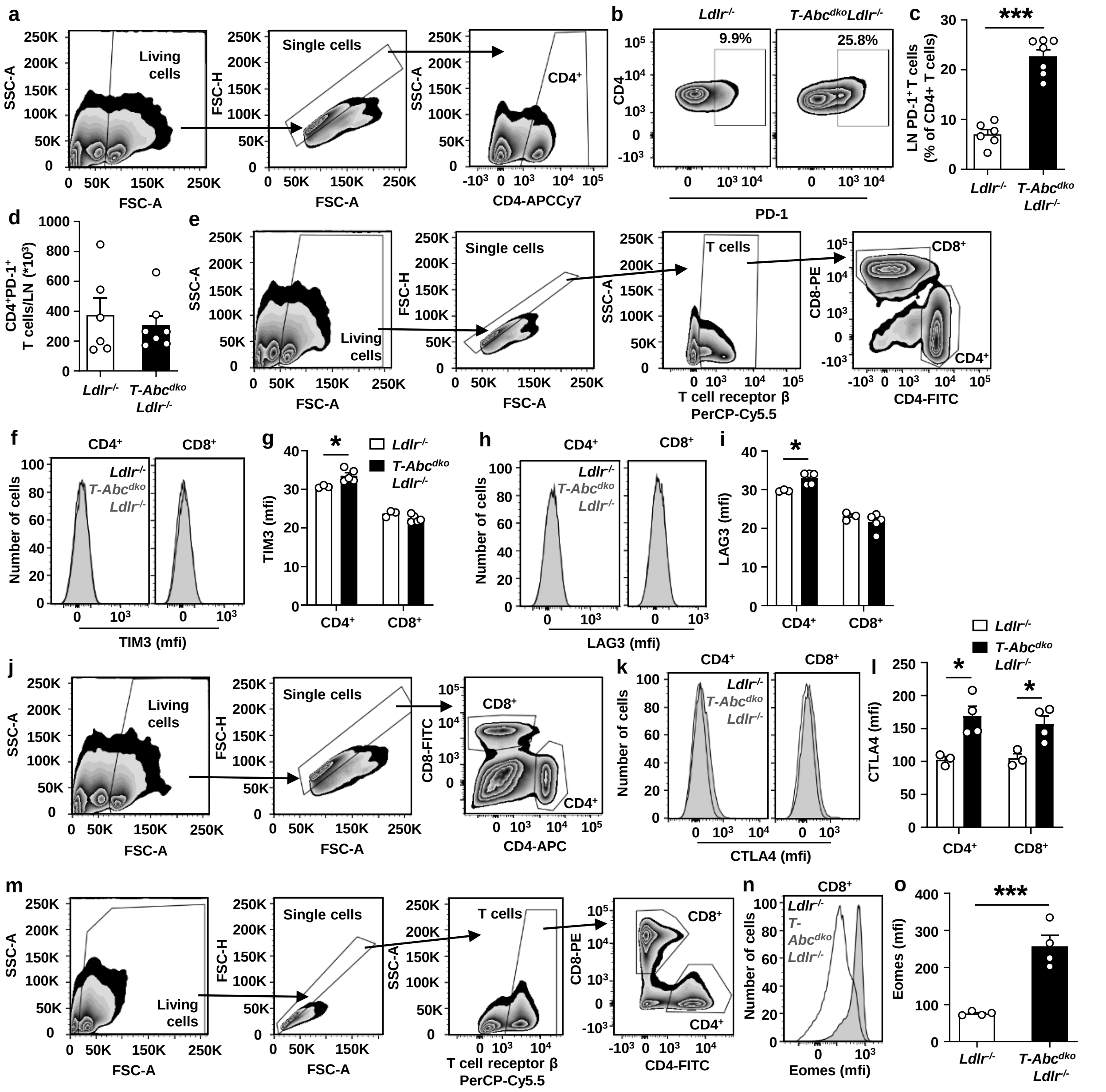

Supplementary Fig. 9 Effects of T cell *Abca1/Abcg1* deficiency on T cell exhaustion in para-aortic LNs. Para-aortic LNs from $Ldlr^{-/-}$ and $T\text{-}Abc^{dko}Ldlr^{-/-}$ mice fed a chow diet were isolated, mashed, and stained with the indicated antibodies and analyzed by flow cytometry. (**a-d**) Programmed cell death protein 1 (PD-1) staining. (**a**) Gating strategy, (**b**) representative flow cytometry plots, and (**c**) quantification of CD4$^+$PD-1$^+$ T cells (p=0.0000019) as a percentage of total para-aortic LN cells, and (**d**) expressed as cells/LN after correction for total para-aortic LN cell number. n=6 $Ldlr^{-/-}$ and n=7 $T\text{-}Abc^{dko}Ldlr^{-/-}$ mice. (**e-i**) T cell immunoglobulin and mucin-domain containing 3 (TIM3) and lymphocyte-activation gene 3 (LAG3) staining. (**e**) Gating strategy, (**f, h**) representative flow cytometry plots for (**f**) TIM3 or (**h**) LAG3, and quantification of (**g**) TIM3 on CD4$^+$ (p=0.026) and CD8$^+$ T cells or (**i**) LAG3 on CD4$^+$ (p=0.011) and CD8$^+$ T cells. n=3 $Ldlr^{-/-}$ and n=5 $T\text{-}Abc^{dko}Ldlr^{-/-}$ mice. (**j-l**) Cytotoxic T-lymphocyte-associated protein 4 (CTLA4) staining. (**j**) Gating strategy, (**k**) representative flow cytometry plots, and (**l**) quantification of CTLA4 on CD4$^+$ (p=0.016) and CD8$^+$ T cells (p=0.022). n=3 $Ldlr^{-/-}$ and n=4 $T\text{-}Abc^{dko}Ldlr^{-/-}$ mice. (**m-o**)

Eomesodermin (Eomes) staining. (**m**) Gating strategy, (**n**) representative flow cytometry plots, and (**o**) quantification of Eomes in CD8$^+$ T cells (p=0.00079). n=4 *Ldlr*$^{-/-}$ and n=4 *T-Abc*^{dko}*Ldlr*$^{-/-}$ mice. For all panels, error bars represent SEM, and biologically independent samples were included. p value was determined by unpaired two-tailed Student's t-test. *p<0.05, ***p<0.001. Source data are provided as a Source Data file.

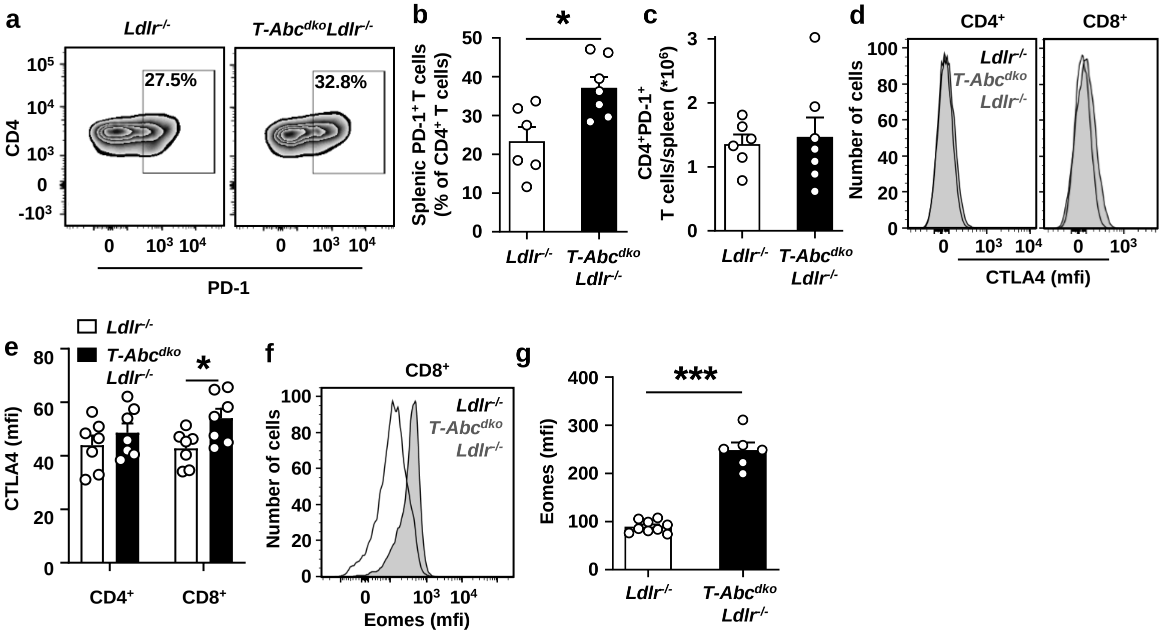

Supplementary Fig. 10 Effects of T cell *Abca1/Abcg1* deficiency on T cell exhaustion in spleen. Spleens from *Ldlr*$^{-/-}$ and *T-Abc*^{dko}*Ldlr*$^{-/-}$ mice fed a chow diet were isolated, mashed, and stained with the indicated antibodies and analyzed by flow cytometry. Gating strategies are shown in Supplemental Fig. 9. (**a-c**) Representative flow cytometry plots of (**a**) CD4$^+$PD-1$^+$ T cells, (**b**) quantification of CD4$^+$PD-1$^+$ T cells (p=0.012) as a percentage of CD4$^+$ T cells, and (**c**) CD4$^+$PD-1$^+$ T cells expressed as cells/spleen after correction for total splenic cell number. n=6 *Ldlr*$^{-/-}$ and n=7 *T-Abc*^{dko}*Ldlr*$^{-/-}$ mice. (**d-g**) Representative flow cytometry plots of (**d**) CTLA4 on CD4$^+$ and CD8$^+$ (p=0.022) T cells and (**f**) Eomes in CD8$^+$ T cells (p<0.000001) and (**e, g**) quantification. (**e**) n=7 *Ldlr*$^{-/-}$ and n=7 *T-Abc*^{dko}*Ldlr*$^{-/-}$ mice. (**g**) n=9 *Ldlr*$^{-/-}$ and n=6 *T-Abc*^{dko}*Ldlr*$^{-/-}$ mice. For all panels, error bars represent SEM, and biologically independent samples were included. p value was determined by unpaired two-tailed Student's t-test. *p<0.05, ***p<0.001. Source data are provided as a Source Data file.

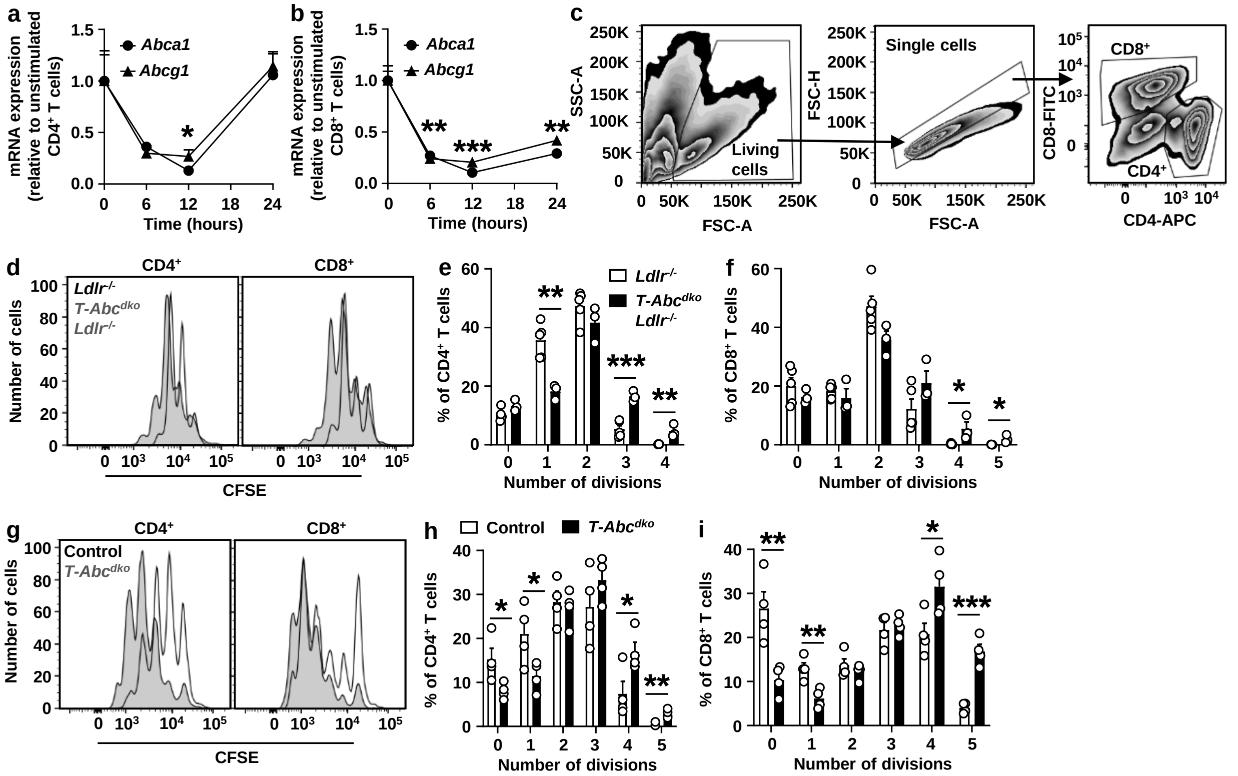

Supplementary Fig. 11 T cell *Abca1/Abcg1* deficiency increases T cell proliferation independent of *Ldlr* expression. Spleens were collected from *Ldlr*$^{-/-}$, *T-Abc*^{dko}*Ldlr*$^{-/-}$, control, and *T-Abc*^{dko} mice fed a chow diet and CD4$^+$ and CD8$^+$ T cells were isolated. (**a-b**) CD4$^+$ and CD8$^+$ T cells were stimulated with αCD3/αCD28 beads for the indicated time. RNA was extracted and *Abca1* and *Abcg1* mRNA expression were measured in (**a**) CD4$^+$ (*Abca1*, p=0.017; *Abcg1*, p=0.022) and (**b**) CD8$^+$ T cells by qPCR (*Abca1* at 6 h, p=0.0012; *Abca1* at 12 h, p=0.00023; *Abca1* at 24 h, p=0.0011; *Abcg1* at 6 h, p=0.00008; *Abcg1* at 12 h, p=0.00004; *Abcg1* at 24 h, p=0.00024). n=5 *Ldlr*$^{-/-}$ mice for time-points 0, 12, and 24 h and n=4 *Ldlr*$^{-/-}$ for time-point 6 h. (**c-i**) T cells were labeled with CFSE and stimulated with αCD3/αCD28 beads. CFSE dilution was measured by flow cytometry at 72 h after stimulation. (**c**) Gating strategy and (**d, g**) representative plots of CFSE dilutions. (**e-f**) The number of divisions were quantified for (**e**) CD4$^+$ (division 1, p=0.0029; 3, p=0.00086; 4, p=0.0029) and (**f**) CD8$^+$ T cells (division 4, p=0.024; 5, p=0.014). n=5 *Ldlr*$^{-/-}$ and n=3 *T-Abc*^{dko}*Ldlr*$^{-/-}$ mice. The experiment was performed twice with the same results. (**h-i**) The number of divisions were quantified for (**h**) CD4$^+$ (division 0, p=0.037; 1, p=0.046; 4, p=0.039; 5, p=0.0026) and (**i**) CD8$^+$ T cells (division 0, p=0.0083; 1, p=0.007; 4, p=0.042; 5, p=0.0003). n=4 control and n=4 *T-Abc*^{dko} mice. For all panels, error bars represent SEM, and biologically independent samples were included. p value was determined by unpaired two-tailed Student's t-test. *p<0.05, **p<0.01, ***p<0.001. Source data are provided as a Source Data file.

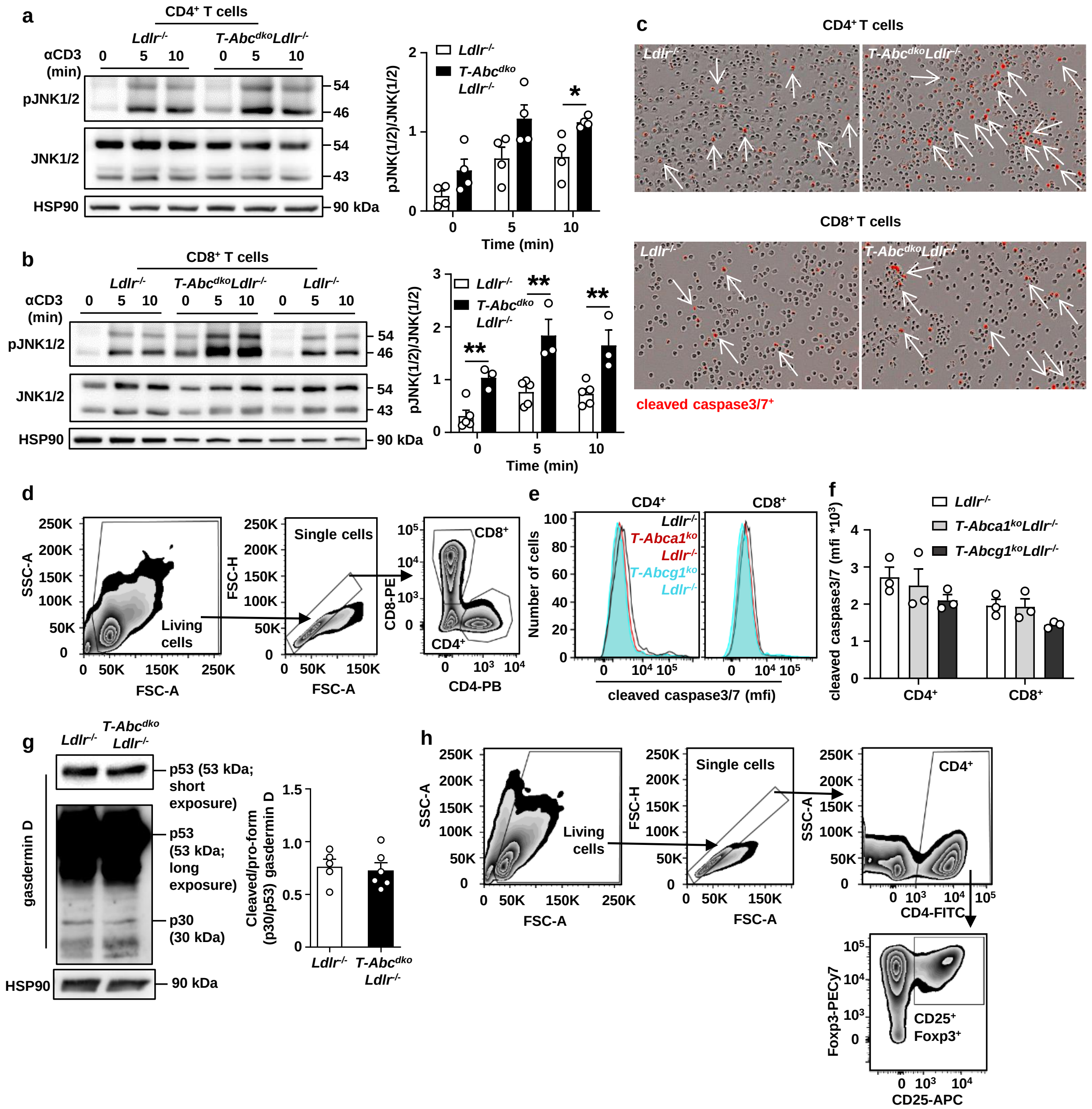

Supplementary Fig. 12 Effects of T cell *Abca1/Abcg1* deficiency on responses downstream of the T cell receptor. Splenic CD4$^+$ and CD8$^+$ T cells were isolated from *Ldlr*$^{-/-}$, *T-Abc*^{dko}*Ldlr*$^{-/-}$, *T-Abca1*^{sko}*Ldlr*$^{-/-}$, and *T-Abcg1*^{sko}*Ldlr*$^{-/-}$ mice fed a chow diet. (**a-b**) T cells were stimulated with αCD3 for the indicated time. (**a**) CD4$^+$ and (**b**) CD8$^+$ T cell lysates were analyzed by Western blot for Jun N-terminal kinase (JNK)1/2 phosphorylation (pJNK1/2) and total JNK1/2. Blots and quantification of the ratio of pJNK1/2 to JNK1/2 for (**a**) CD4$^+$ (p=0.019) and (**b**) CD8$^+$ (0 min, p=0.0055; 5 min, p=0.0068; 10 min, p=0.0099) T cells. Heat shock protein (HSP)90 was used as loading control. (**a**) n=4 *Ldlr*$^{-/-}$ and n=4 *T-Abc*^{dko}*Ldlr*$^{-/-}$ mice. (**b**) n=5 *Ldlr*$^{-/-}$ and n=3 *T-Abc*^{dko}*Ldlr*$^{-/-}$ mice. (**c-f**) T cells were stimulated with αCD3 and interleukin (IL)-2 for 12 h with concomitant staining for cleaved caspase3/7. (**c**) CD4$^+$ and CD8$^+$ T cells acquiring cleaved caspase 3/7 staining over time were assessed using the Incucyte system and are depicted by arrows in representative snapshots taken at 10 h after stimulation. (**d**) Gating strategy, (**e**) representative

flow cytometry plots, and (**f**) quantification of cleaved caspase 3/7 in T cells. n=3 *Ldlr*$^{-/-}$, n=3 *T-Abca1*^{sko}*Ldlr*$^{-/-}$, and n=3 *T-Abcg1*^{sko}*Ldlr*$^{-/-}$ mice. (**g**) CD4^{+} T cells were stimulated with αCD3/IL-2 for 12 h, lysed, and analyzed by Western blot for gasdermin D cleavage. Blot and quantification of the ratio of cleaved gasdermin D p30 to gasdermin D p53 (pro-form). HSP90 was used as loading control. n=5 *Ldlr*$^{-/-}$ and n=6 *T-Abc*^{dko}*Ldlr*$^{-/-}$ mice. (**a-b, g**) On the same blot, equal sample numbers from *Ldlr*$^{-/-}$ and *T-Abc*^{dko}*Ldlr*$^{-/-}$ mice were loaded. In parallel, more blots derived from the same experiment were run, processed, and added for quantification. (**h**) Gating strategy for CD25^{+}Foxp3^{+} T_{reg} cells after stimulation with αCD3/αCD28 beads, IL-2, and transforming growth factor β (TGF-β) for 72 h. For all panels, error bars represent SEM. Biologically independent samples were included. *p* value was determined by unpaired two-tailed Student's t-test (**a-b, g**) or one-way ANOVA with Bonferroni post-test (**f**). **p*<0.05, ***p*<0.01. Source data are provided as a Source Data file.

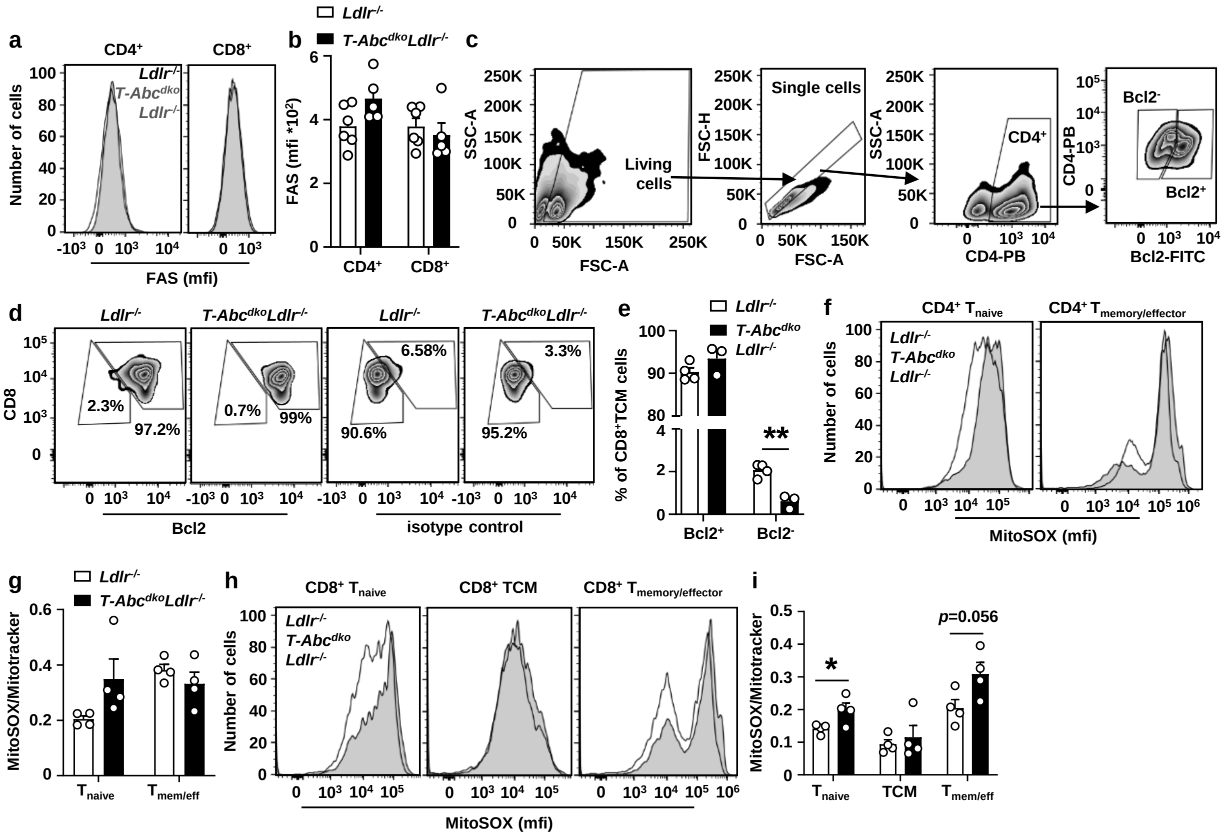

Supplementary Fig. 13 Effects of T cell *Abca1/Abcg1* deficiency on pathways regulating apoptosis downstream of the T cell receptor. Spleens were collected from *Ldlr*$^{-/-}$ and *T-Abc*^{dko}*Ldlr*$^{-/-}$ mice fed a chow diet and CD4^{+} and CD8^{+} T cells were isolated. (**a-i**) CD4^{+} and CD8^{+} T cells were stimulated with αCD3 and IL-2 for 12 h, stained for FAS (**a-b**), B-cell lymphoma 2 (Bcl2) (**c-e**), or a combination of MitoSOX and MitoTracker Green (**f-i**). (**a-b**) Representative flow cytometry plots of (**a**) FAS expression on CD4^{+} and CD8^{+} T cells gated as in Supplementary Fig. 12d and (**b**) quantification. n=6 *Ldlr*$^{-/-}$ and n=5 *T-Abc*^{dko}*Ldlr*$^{-/-}$ mice. (**c**) Gating strategy for CD4^{+}Bcl2^{+} and Bcl2^{-} T cells. (**d-e**) Representative flow cytometry plots of (**d**) Bcl2^{+} and Bcl2^{-} (*p*=0.0018) CD8^{+} TCM cells gated as in Supplementary Fig. 8a, (**e**) corrected for their isotype control, and quantified. n=4 *Ldlr*$^{-/-}$ and n=3 *T-Abc*^{dko}*Ldlr*$^{-/-}$ mice. (**f, h**) Representative flow cytometry plots of MitoSOX levels in (**f**) CD4^{+} T_{naive} and $T_{mem/eff}$ cells and (**h**) CD8^{+} T_{naive}, $T_{mem/eff}$, and TCM cells. (**g, i**) Quantification of MitoSOX levels in (**g**) CD4^{+} T_{naive} and $T_{mem/eff}$ cells and (**i**) CD8^{+} T_{naive} (*p*=0.044), $T_{mem/eff}$, and TCM cells. n=4 *Ldlr*$^{-/-}$ and n=4 *T-Abc*^{dko}*Ldlr*$^{-/-}$ mice. MitoSOX values in (**c**) and (**e**) have been corrected for MitoTracker Green levels. For all panels, error bars represent SEM, and biologically independent samples were included. *p* value was determined by unpaired two-tailed Student's t-test. *$p<0.05$, **$p<0.01$. Source data are provided as a Source Data file.

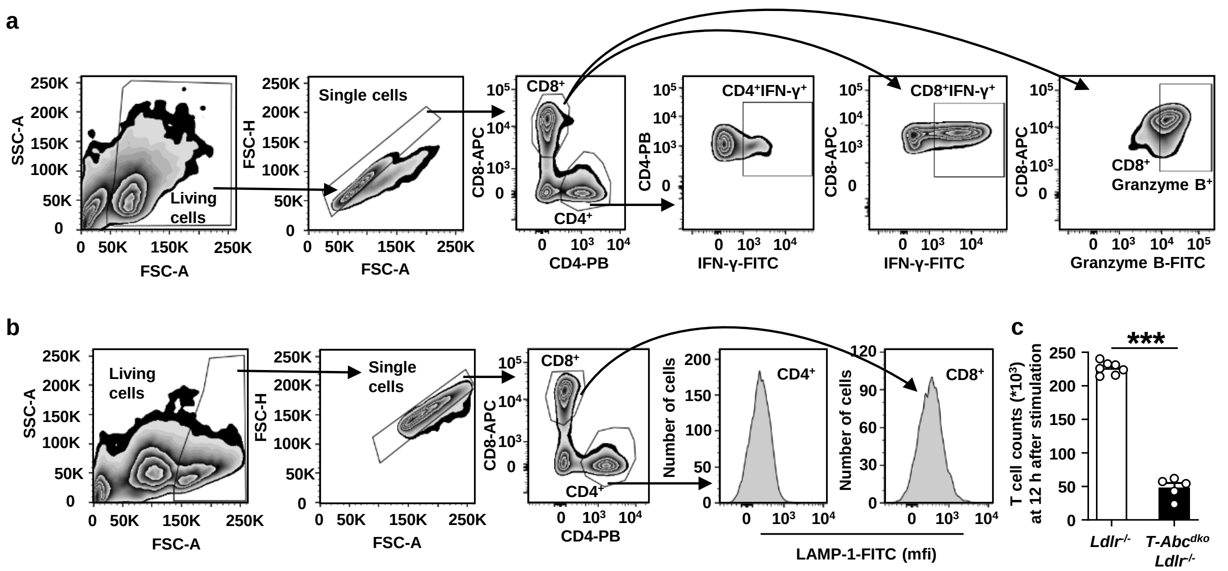

Supplementary Fig. 14 Gating strategies for markers of T cell functionality and T cell counts upon T cell receptor stimulation. Spleens were collected from *Ldlr*$^{-/-}$ and *T-Abc*^{dko}*Ldlr*$^{-/-}$ mice fed a chow diet and CD4^{+} and CD8^{+} T cells were isolated. (**a-b**) CD4^{+} and CD8^{+} T cells were stimulated with αCD3/IL-2 for 12 h, fixed and permeabilized, stained for interferon gamma (IFN-γ) or granzyme B (**a**), or, alternatively, not fixed, and stained for lysosomal-associated membrane protein-1 (LAMP-1) (**b**). (**a-b**) Gating strategy for (**a**) CD4^{+}IFN-γ^{+}, CD8^{+}IFN-γ^{+}, and CD8^{+}granzymeB^{+} T cells and (**b**) expression of LAMP-1 on CD4^{+} and CD8^{+} T cells. (**c**) T cells (300,000 T cells per mouse of each genotype) were isolated and stimulated with αCD3 and IL-2 for 12 h, harvested, and counted (p<0.000001). n=7 *Ldlr*$^{-/-}$ and n=5 *T-Abc*^{dko}*Ldlr*$^{-/-}$ mice. Error bars represent SEM, and biologically independent samples were included. p value was determined by unpaired two-tailed Student's t-test. ***p<0.001. Source data are provided as a Source Data file.

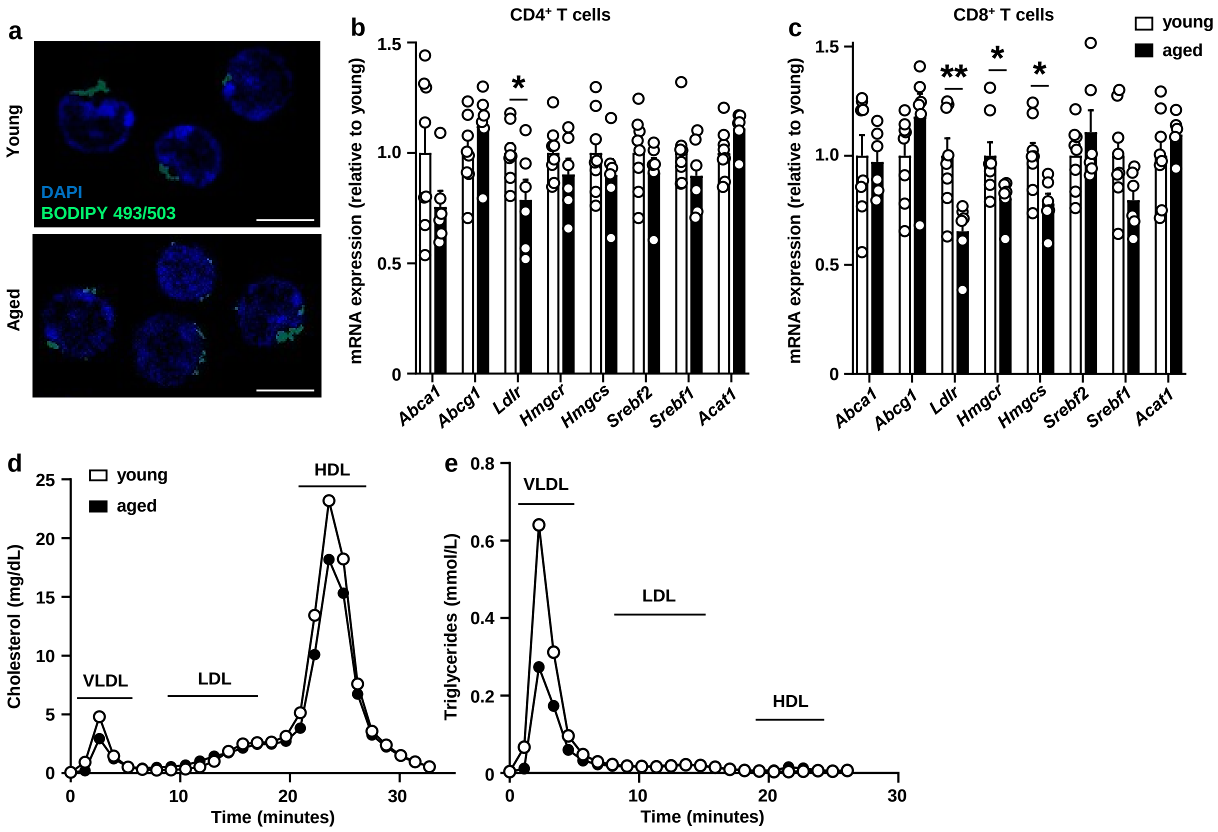

Supplementary Fig. 15 Effects of aging on lipids in T cells and plasma lipids in wild-type mice. Spleens and blood from young (3 months) and aged (24 months) wild-type mice fed a chow diet were collected. (**a**) Splenic $CD4^+$ and $CD8^+$ T cells were fixed, permeabilized, and stained with BODIPY 493/503 and DAPI. Samples were analyzed by confocal microscopy and representative pictures are shown. Per mouse, 20 T cells were analyzed. n=3 young and n=3 aged wild-type mice. Scale bar represents 5 µm. (**b-c**) Splenic (**b**) $CD4^+$ and (**c**) $CD8^+$ T cells were isolated and RNA was extracted. *Abca1, Abcg1, Ldlr* ($CD4^+$, *p*=0.042; $CD8^+$, *p*=0.0063)*, Hmgcr* (*p*=0.029)*, Hmgcs* (*p*=0.0165)*, Srebf2, Srebf1, and Acat1* mRNA expression were measured by qPCR. n=8 young and n=6 aged wild-type mice. For (**b-c**), error bars represent SEM, and biologically independent samples were included. *p* value was determined by unpaired two-tailed Student's t-test. **p*<0.05, ***p*<0.01. (**d-e**) Plasma samples were pooled (n=16 young and n=16 aged wild-type mice per pool), separated using fast performance liquid chromatography (FPLC), and total cholesterol (**d**) and triglyceride (**e**) levels were determined. VLDL, very low-density lipoprotein; LDL, low-density lipoprotein; HDL high-density lipoprotein. Source data are provided as a Source Data file.

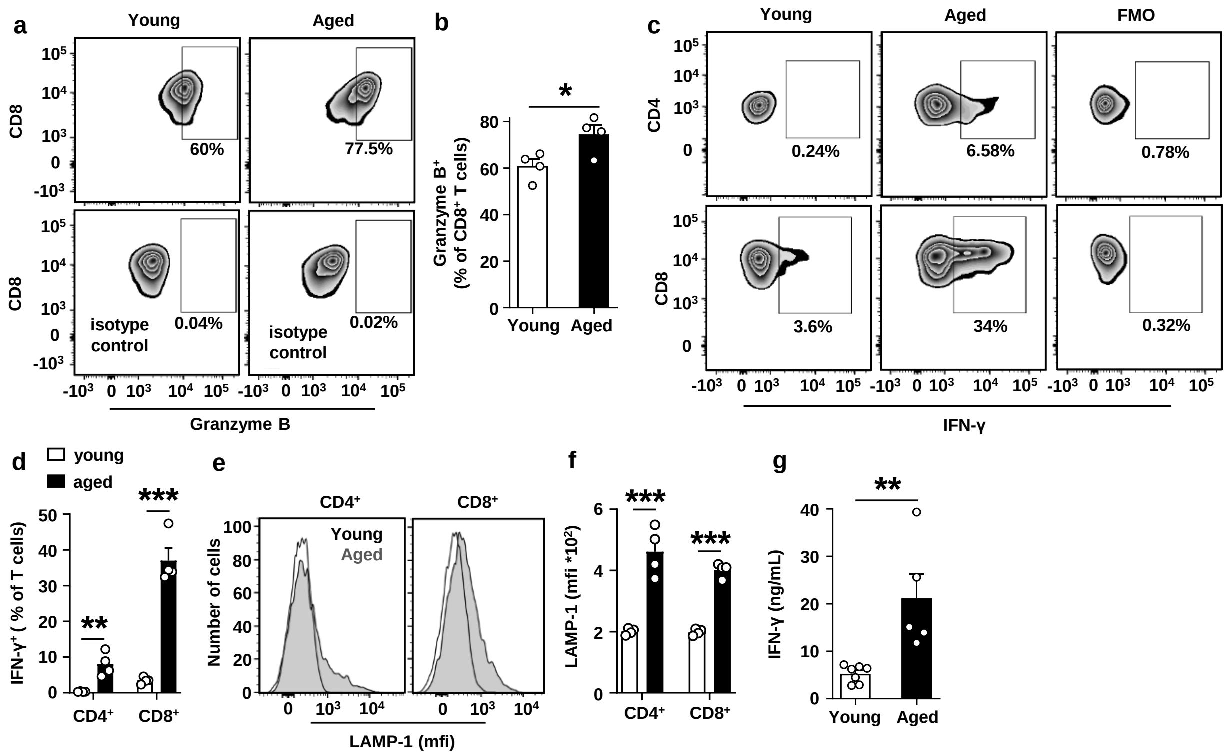

Supplementary Fig. 16 Aging increases markers of T cell functionality in wild-type mice. Spleens and blood from young (3 months) and aged (24 months) wild-type mice fed a chow diet were collected. (**a-f**) $CD4^+$ and $CD8^+$ T cells were isolated and stimulated with αCD3 and IL-2. After 12 h, cells were fixed and permeabilized and stained for granzyme B (**a-b**) or IFN-γ (**c-d**), or, alternatively, not fixed, and stained for LAMP-1 (**e-f**). Gating strategies are shown in Supplemental Fig. 14. (**a-b**) Representative flow cytometry plots of (**a**) $CD8^+$granzyme B^+ T cells and (**b**) quantification (p=0.0032) as a percentage of total $CD8^+$ T cells after correction for isotype control. n=4 young and n=4 aged wild-type mice. (**c-d**) Representative flow cytometry plots of (**c**) $CD4^+$IFN-γ^+ and $CD8^+$IFN-γ^+ cells and (**d**) quantification as a percentage of $CD4^+$ (p=0.0036) or $CD8^+$ (p=0.00008) T cells after correction for fluorescence minus one (FMO) control. n=4 young and n=4 aged wild-type mice. (**e-f**) Representative flow cytometry plots of (**e**) LAMP-1 expression on $CD4^+$ (p=0.0006) and $CD8^+$ (p=0.000004) T cells and (**f**) quantification. n=4 young and n=4 aged wild-type mice. (**g**) Total T cells were stimulated with αCD3/IL-2 for 12 h. Medium was collected and IFN-γ levels (p=0.0043) were measured by ELISA. n=7 young and n=5 aged wild-type mice. For all panels, error bars represent SEM, and biologically independent samples were included. p value was determined by unpaired two-tailed Student's t-test. *p<0.05, **p<0.01, ***p<0.001. Source data are provided as a Source Data file.

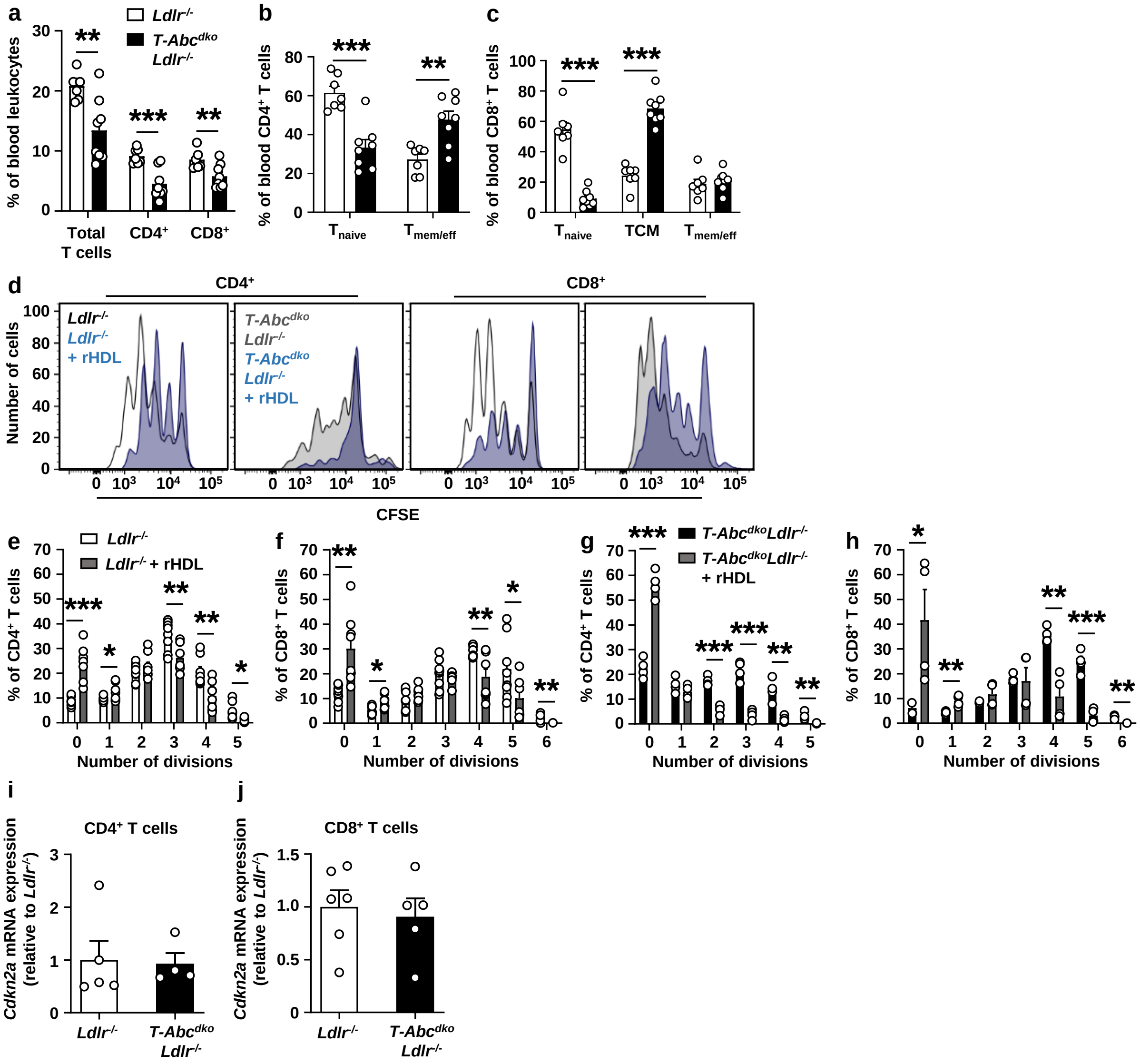

Supplementary Fig. 17 T cell *Abca1/Abcg1* deficiency decreases blood T cells, increases T cell activation, and decreases T cell proliferation in middle-aged *Ldlr*$^{-/-}$ mice with further reduction by reconstituted high-density lipoprotein. Blood and spleens from *Ldlr*$^{-/-}$ and *T-Abc*^{dko}*Ldlr*$^{-/-}$ mice fed a chow diet for 12-13 months were collected. (**a**) Total (p=0.0048), CD4^{+} (p=0.0007), and CD8^{+} (p=0.0104) T cells as a percentage of total leukocytes. n=7 *Ldlr*$^{-/-}$ and n=8 *T-Abc*^{dko}*Ldlr*$^{-/-}$ mice. (**b-c**) CD4^{+} T_{naive} (p=0.0002) and $T_{mem/eff}$ (p=0.0019) (**b**), and CD8^{+} T_{naive} (p<0.000001), $T_{mem/eff}$, and TCM (p<0.000001) (**c**) as a percentage of total CD4^{+} (**b**) or CD8^{+} (**c**) T cells in blood. n=7 *Ldlr*$^{-/-}$ and n=8 *T-Abc*^{dko}*Ldlr*$^{-/-}$ mice. (**d-h**) Splenic T cells were labeled with CFSE, incubated with or without reconstituted high-density lipoprotein (rHDL), and stimulated with αCD3/αCD28 beads. CFSE dilution was measured by flow cytometry at 72 h after stimulation. (**d**) Representative CFSE dilutions in CD4^{+} and CD8^{+} T cells gated as in Supplementary Fig. 11c. The number of divisions were quantified for (**e**) CD4^{+} *Ldlr*$^{-/-}$ (division 0, p=0.000023; 1, p=0.039; 3, p=0.0061; 4, p=0.0033; 5, p=0.014) and (**g**) CD4^{+} *T-Abc*^{dko}*Ldlr*$^{-/-}$ (division 0, p=0.000057; 2, p=0.00012; 3, p=0.00019; 4, p=0.0027; 5, p=0.0039) T cells and (**f**) CD8^{+} *Ldlr*$^{-/-}$ (division 0, p=0.0029; 1, p=0.0177; 4, p=0.0089; 5, p=0.0418; 6, p=0.0043) and (**h**) CD8^{+} *T-Abc*^{dko}*Ldlr*$^{-/-}$ (division 0, p=0.0287; 1, p=0.0057; 4, p=0.0019; 5, p=0.00017; 6, p=0.0029) T cells. T cells from

n=8 *Ldlr*$^{-/-}$ and n=8 *T-Abc*^{dko}*Ldlr*$^{-/-}$ mice were incubated with or without rHDL. (**i-j**) Splenic T cells were isolated and RNA was extracted. Cyclin dependent kinase inhibitor 2a (*Cdkn2a*) mRNA expression was measured in (**i**) CD4^{+} and (**j**) CD8^{+} T cells by qPCR. (**i**) n=5 *Ldlr*$^{-/-}$ and n=4 *T-Abc*^{dko}*Ldlr*$^{-/-}$ mice. (**j**) n=6 *Ldlr*$^{-/-}$ and n=5 *T-Abc*^{dko}*Ldlr*$^{-/-}$ mice. For all panels, error bars represent SEM, and biologically independent samples were included. *p* value was determined by unpaired two-tailed Student's t-test. *$p<0.05$, **$p<0.01$, ***$p<0.001$. Source data are provided as a Source Data file.

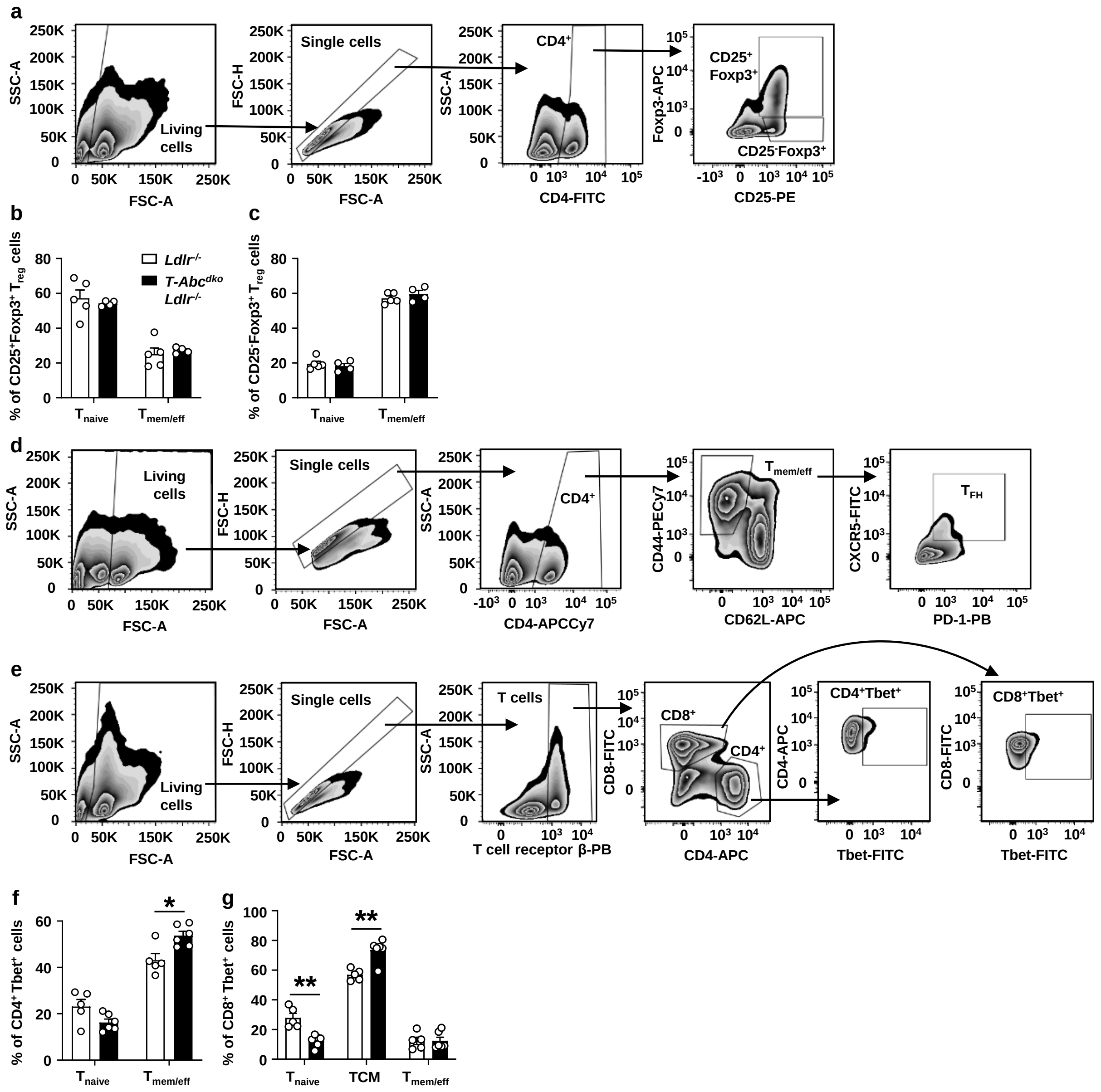

Supplementary Fig. 18 Effects of T cell *Abca1/Abcg1* deficiency on LN T cell subsets in *Ldlr*$^{-/-}$ mice. Female *Ldlr*$^{-/-}$ and *T-Abc*^{dko}*Ldlr*$^{-/-}$ mice were fed chow diet for 28 weeks. Para-aortic LNs were collected, mashed, stained with the indicated antibodies, and analyzed by flow cytometry. (**a**) Gating strategy for CD25$^-$Foxp3$^+$ and CD25$^+$Foxp3$^+$ T$_{reg}$ cells. (**b-c**) T$_{naive}$ and T$_{mem/eff}$ cells gated as in Supplementary Fig. 8a were quantified as a percentage of (**b**) CD25$^+$Foxp3$^+$ and (**c**) CD25$^-$Foxp3$^+$ T$_{reg}$ cells. n=5 *Ldlr*$^{-/-}$ and n=4 *T-Abc*^{dko}*Ldlr*$^{-/-}$ mice. (**d**) Gating strategy for CD4$^+$CD44$^+$CD62L$^-$C-X-C chemokine receptor type 5 (CXCR5)$^+$PD-1$^+$ T$_{follicular\ helper}$ (T$_{FH}$) cells. (**e**) Gating strategy for CD4$^+$Tbet$^+$ and CD8$^+$Tbet$^+$ cells. (**f-g**) T$_{naive}$ and T$_{mem/eff}$ (p=0.0102) cells gated as in Supplementary Fig. 8a were quantified as a percentage of CD4$^+$Tbet$^+$ (**f**) and T$_{naive}$ (p=0.0011), T$_{mem/eff}$, and TCM (p=0.0014) cells gated as in Supplementary Fig. 8a were quantified as a percentage of CD8$^+$Tbet$^+$ (**g**) cells. n=5 *Ldlr*$^{-/-}$ and n=6 *T-Abc*^{dko}*Ldlr*$^{-/-}$ mice. For all panels, error bars represent SEM, and biologically independent samples were included. p value was determined by unpaired two-tailed Student's t-test. *p<0.05, **p<0.01. Source data are provided as a Source Data file.

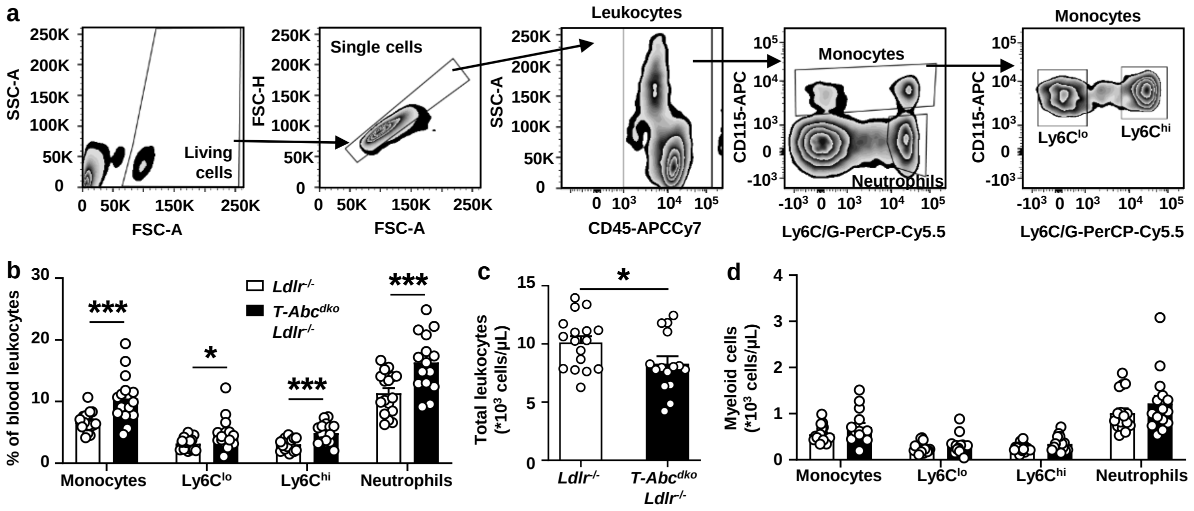

Supplementary Fig. 19 T cell *Abca1/Abcg1* deficiency does not affect blood myeloid cell numbers in *Ldlr*$^{-/-}$ mice fed chow diet. Blood was collected from female *Ldlr*$^{-/-}$ and *T-Abc*^{dko}*Ldlr*$^{-/-}$ mice fed a chow diet for 28 weeks. Leukocytes were stained with the indicated antibodies and analyzed by flow cytometry. (**a**) Gating strategy for monocytes (CD45^{+}CD115^{+}), Ly6C^{lo} and Ly6C^{hi} monocyte subsets, and neutrophils (CD45^{+}CD115^{-}Ly6G^{+}). (**b**) Monocytes (*p*=0.0010), Ly6C^{lo} (*p*=0.0234) and Ly6C^{hi} (*p*=0.0006) monocyte subsets, and neutrophils (*p*=0.0015) as a percentage of leukocytes. n=17 *Ldlr*$^{-/-}$ and n=15 *T-Abc*^{dko}*Ldlr*$^{-/-}$ mice. (**c**) Total blood leukocyte counts (*p*=0.036). n=17 *Ldlr*$^{-/-}$ and n=15 *T-Abc*^{dko}*Ldlr*$^{-/-}$ mice. (**d**) Concentration of myeloid cells in blood. Percentages of myeloid cells as in **b** were corrected for total leukocyte numbers as in **d**. n=17 *Ldlr*$^{-/-}$ and n=15 *T-Abc*^{dko}*Ldlr*$^{-/-}$ mice. For all panels, error bars represent SEM, and biologically independent samples were included. *p* value was determined by unpaired two-tailed Student's t-test. *$p<0.05$, ***$p<0.001$. Source data are provided as a Source Data file.

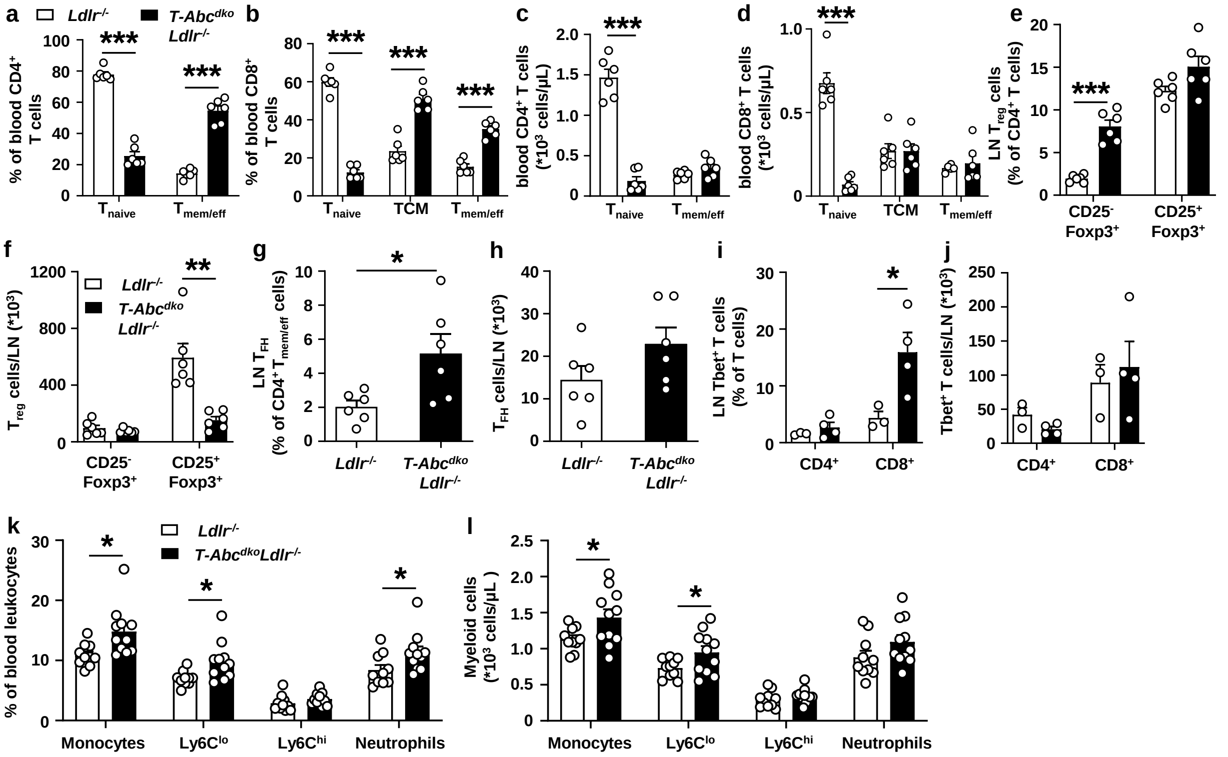

Supplementary Fig. 20 Effects of T cell *Abca1/Abcg1* deficiency on naïve and activated T cells and myeloid cells in blood and LN T cell subsets in *Ldlr$^{-/-}$* mice fed Western-type diet. Female *Ldlr$^{-/-}$* and *T-Abc^{dko}Ldlr$^{-/-}$* mice were fed a Western-type diet (WTD) for 10 weeks. Blood and para-aortic LNs were collected, cells were stained with the indicated antibodies, and analyzed by flow cytometry. (**a-b**) CD4$^+$ T$_{naive}$ and T$_{mem/eff}$ (*p*<0.000001) (**a**) and CD8$^+$ T$_{naive}$ (*p*<0.000001), T$_{mem/eff}$ (*p*=0.0006), and TCM (*p*=0.000015) (**b**) as a percentage of blood (**a**) CD4$^+$ and (**b**) CD8$^+$ T cells. The experiment was performed twice with the same results. (**c-d**) CD4$^+$ T$_{naive}$ (*p*<0.000001) and T$_{mem/eff}$ (**c**) and CD8$^+$ T$_{naive}$ (*p*=0.0000027), T$_{mem/eff}$, and TCM (**d**) corrected for total leukocytes. n=6 *Ldlr$^{-/-}$* and n=6 *T-Abc^{dko}Ldlr$^{-/-}$* mice. (**e-f**) CD25$^-$Foxp3$^+$ (*p*=0.000011) and CD25$^+$Foxp3$^+$ T$_{reg}$ cells as a percentage of CD4$^+$ LN T cells, after correction for isotype control (**e**) and CD25$^-$Foxp3$^+$ and CD25$^+$Foxp3$^+$ (*p*=0.0016) T$_{reg}$ cells expressed as cells/LN after correction for total LN cell numbers (**f**). n=6 *Ldlr$^{-/-}$* and n=6 *T-Abc^{dko}Ldlr$^{-/-}$* mice. (**g-h**) T$_{FH}$ cells (*p*=0.025) as a percentage of total CD4$^+$ LN T cells (**g**) and expressed as cells/LN after correction for total LN cell numbers (**h**). n=6 *Ldlr$^{-/-}$* and n=6 *T-Abc^{dko}Ldlr$^{-/-}$* mice. (**i-j**) CD4$^+$Tbet$^+$ and CD8$^+$Tbet$^+$ (*p*=0.041) cells as a percentage of total CD4$^+$ or CD8$^+$ LN T cells, after correction for isotype control (**i**) and expressed as cells/LN (**j**). n=3 *Ldlr$^{-/-}$* and n=4 *T-Abc^{dko}Ldlr$^{-/-}$* mice. (**k-l**) Monocytes (*p*=0.0128), Ly6C^{lo} (*p*=0.0163) and Ly6C^{hi} monocyte subsets, and neutrophils (*p*=0.0382) as a percentage of total blood leukocytes (**k**), and (**l**) monocytes (*p*=0.031), Ly6C^{lo} (*p*=0.044) and Ly6C^{hi} monocyte subsets, and neutrophils after correction for total blood leukocyte counts. n=10 *Ldlr$^{-/-}$* and n=11 *T-Abc^{dko}Ldlr$^{-/-}$* mice. For all panels, error bars represent SEM, and biologically independent samples were included. *p* value was determined by unpaired two-tailed Student's t-test. **p*<0.05, ***p*<0.01, ****p*<0.001. Source data are provided as a Source Data file.

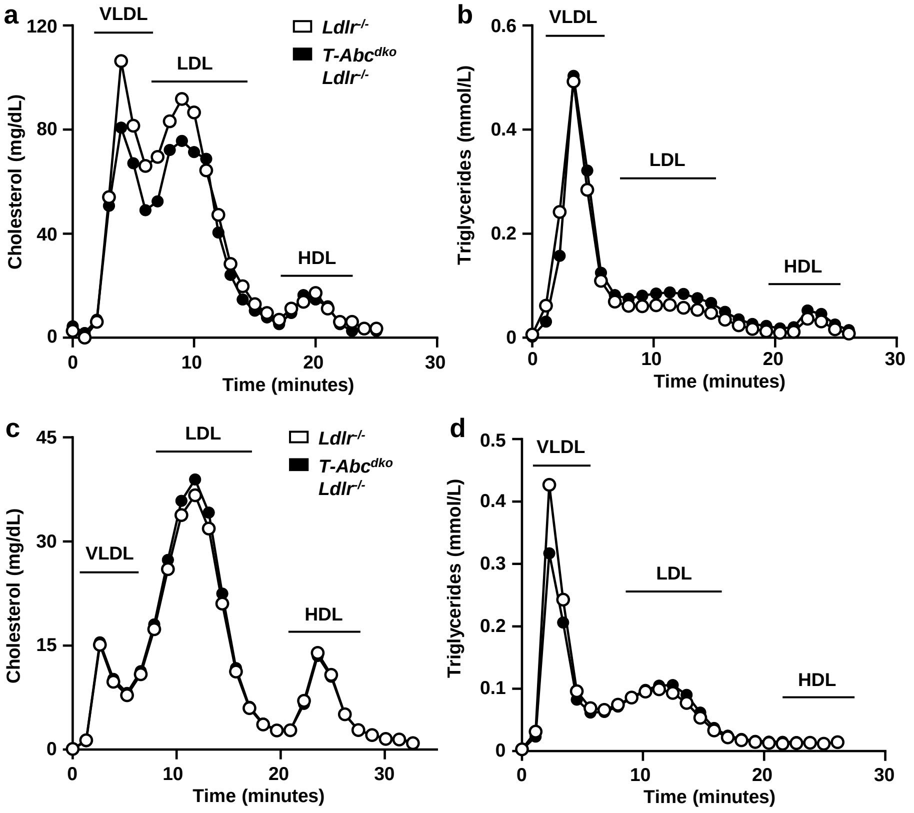

Supplementary Fig. 21 Effects of T cell *Abca1/Abcg1* deficiency on cholesterol and triglyceride lipoprotein profiles in *Ldlr*$^{-/-}$ mice. Female *Ldlr*$^{-/-}$ and *T-Abc*^{dko}*Ldlr*$^{-/-}$ mice were fed a WTD for 10 weeks (**a-b**) or a chow diet for 28 weeks (**c-d**). Lipoproteins from pooled plasma samples of mice fed WTD (**a-b**; n=15 *Ldlr*$^{-/-}$ and n=15 *T-Abc*^{dko}*Ldlr*$^{-/-}$ mice per pool) or chow diet (**c-d**; n=18 *Ldlr*$^{-/-}$ and n=16 *T-Abc*^{dko}*Ldlr*$^{-/-}$ mice per pool) were separated using FPLC and total cholesterol (**a, c**) and triglyceride (**b, d**) levels were determined. Source data are provided as a Source Data file.

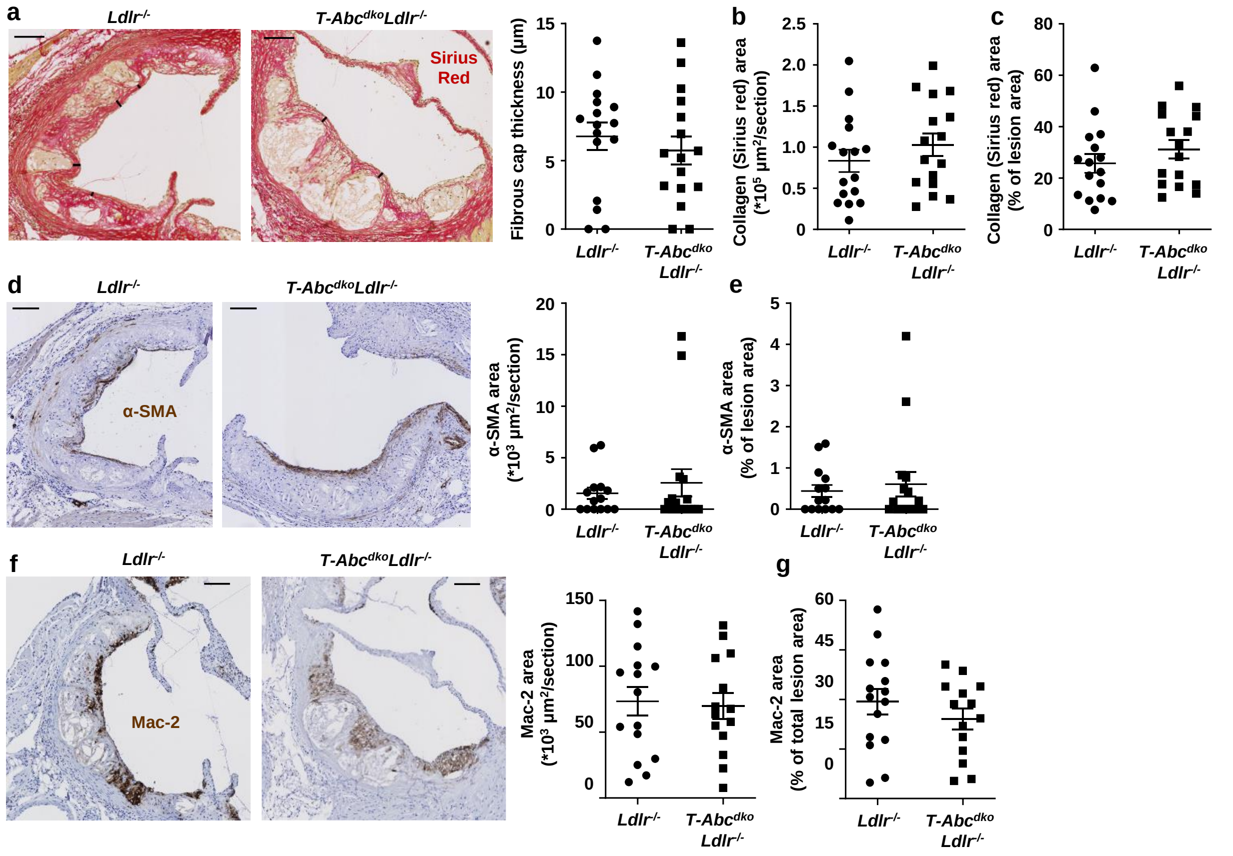

Supplementary Fig. 22 T cell *Abca1/Abcg1* deficiency does not affect atherosclerotic lesion composition in *Ldlr*$^{-/-}$ mice fed a Western-type diet. Female *Ldlr*$^{-/-}$ and *T-Abc*^{dko}*Ldlr*$^{-/-}$ mice were fed a WTD for 10 weeks. Hearts were isolated, sectioned, and paraffin sections of the aortic root were stained for (**a-c**) Sirius Red, (**d-e**) smooth muscle actin (α-SMA), or (**f-g**) Mac-2. (**a**) Representative pictures of Sirius Red staining (left) and quantification (right) of fibrous cap thickness. (**b-c**) Total collagen area (Sirius Red^{+}) (**b**) and collagen area as a percentage of total lesion area (**c**). (**a-c**) n=16 *Ldlr*$^{-/-}$ and n=16 *T-Abc*^{dko}*Ldlr*$^{-/-}$ mice. (**d**) Representative pictures (left) and quantification (right) of total α-SMA area. (**e**) α-SMA area as a percentage of total lesion area. (**d-e**) n=14 *Ldlr*$^{-/-}$ and n=16 *T-Abc*^{dko}*Ldlr*$^{-/-}$ mice. (**f**) Representative pictures (left) and quantification (right) of total Mac-2 area. (**g**) Mac-2 area as a percentage of total lesion area. (**f-g**) n=15 *Ldlr*$^{-/-}$ and n=14 *T-Abc*^{dko}*Ldlr*$^{-/-}$ mice. (**a, d, f**) Scale bar represents 100 µm. For all panels, error bars represent SEM, and biologically independent samples were included. *p* value was determined by unpaired two-tailed Student's t-test. Source data are provided as a Source Data file.

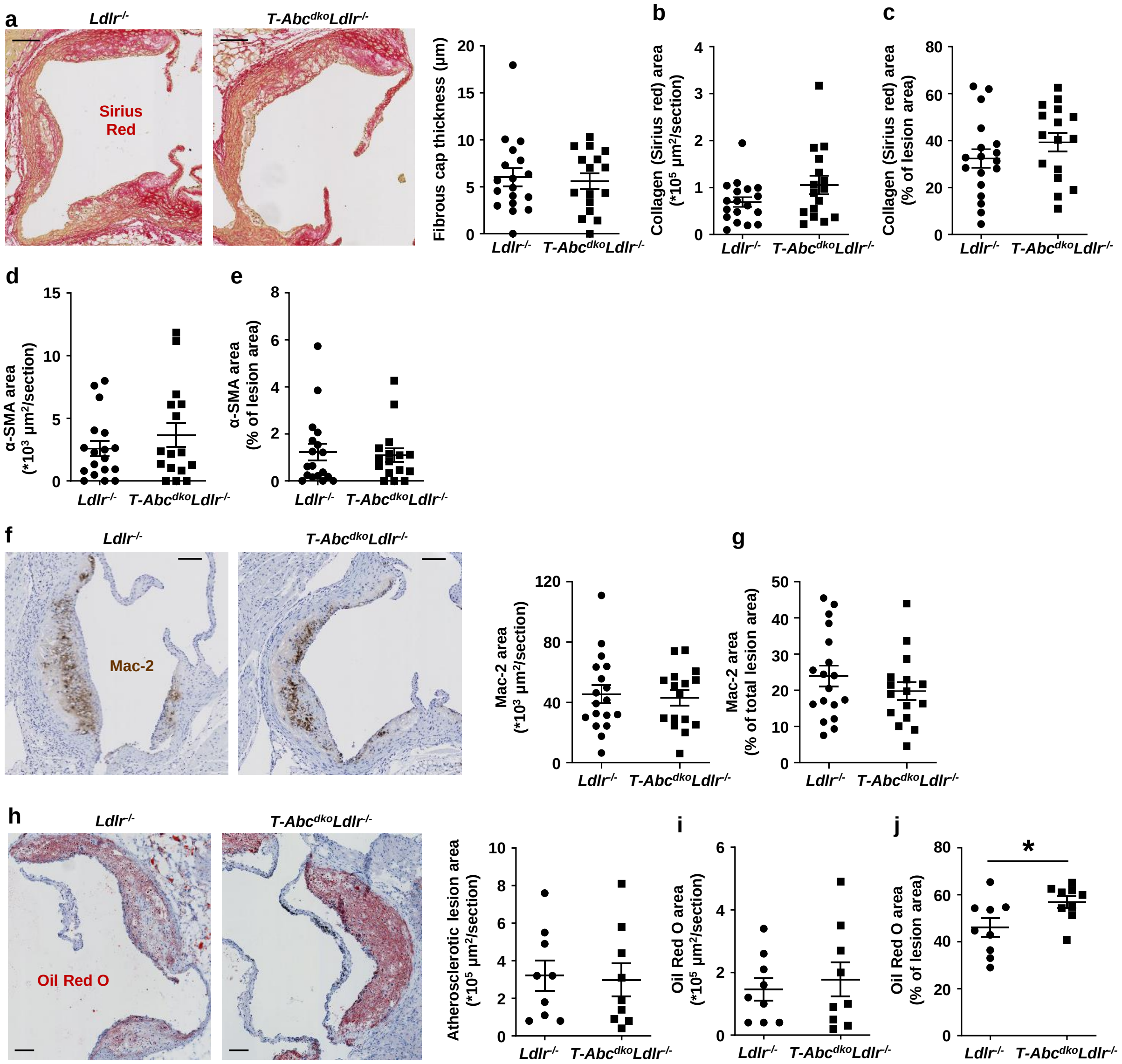

Supplementary Fig. 23 T cell *Abca1/Abcg1* deficiency increases lipid accumulation, but otherwise does not affect atherosclerotic lesion composition in *Ldlr$^{-/-}$* mice fed a chow diet. Female *Ldlr$^{-/-}$* and *T-Abc^{dko}Ldlr$^{-/-}$* mice were fed a chow diet for 28 weeks (**a-g**) or 38 weeks (**h-j**). (**a-g**) Characterization of atherosclerotic lesions of the aortic root from mice fed chow diet for 28 weeks. Hearts were isolated, sectioned, and paraffin sections of the aortic root were stained for (**a-c**) Sirius Red, (**d-e**) smooth muscle actin (α-SMA), or (**f-g**) Mac-2. (**a**) Representative pictures of Sirius Red staining (left) and quantification (right) of fibrous cap thickness. (**b-c**) Total collagen area (Sirius Red^{+}) (**b**) and collagen area as a percentage of total lesion area (**c**). (**a-c**) n=18 *Ldlr$^{-/-}$* and n=16 *T-Abc^{dko}Ldlr$^{-/-}$* mice. (**d-e**) Total α-SMA area (**d**) and α-SMA area as a percentage of total lesion area (**e**). (**d-e**) n=18 *Ldlr$^{-/-}$* and n=16 *T-Abc^{dko}Ldlr$^{-/-}$* mice. (**f**) Representative pictures (left) and quantification (right) of total Mac-2 area. (**g**) Mac-2 area as a percentage of total lesion area. (**f-g**) n=16 *Ldlr$^{-/-}$* and n=18 *T-Abc^{dko}Ldlr$^{-/-}$* mice. (**h-j**) Mice were fed a chow diet for 38 weeks, hearts were isolated, embedded in OCT, sectioned, and frozen sections of the aortic root were stained for Oil Red O. (**h**) Representative pictures of Oil Red O staining (left) and quantification (right) of atherosclerotic lesion area. (**i-j**) Total Oil Red O area (**i**) and Oil

Red O area as a percentage (p=0.0347) of total atherosclerotic lesion area (**j**). (**h-j**) n=9 *Ldlr*$^{-/-}$ and n=9 *T-Abc*^{dko}*Ldlr*$^{-/-}$ mice. (**a, f, h**) Scale bar represents 100 µm. For all panels, error bars represent SEM, and biologically independent samples were included. *p* value was determined by unpaired two-tailed Student's t-test. *$p<0.05$. Source data are provided as a Source Data file.

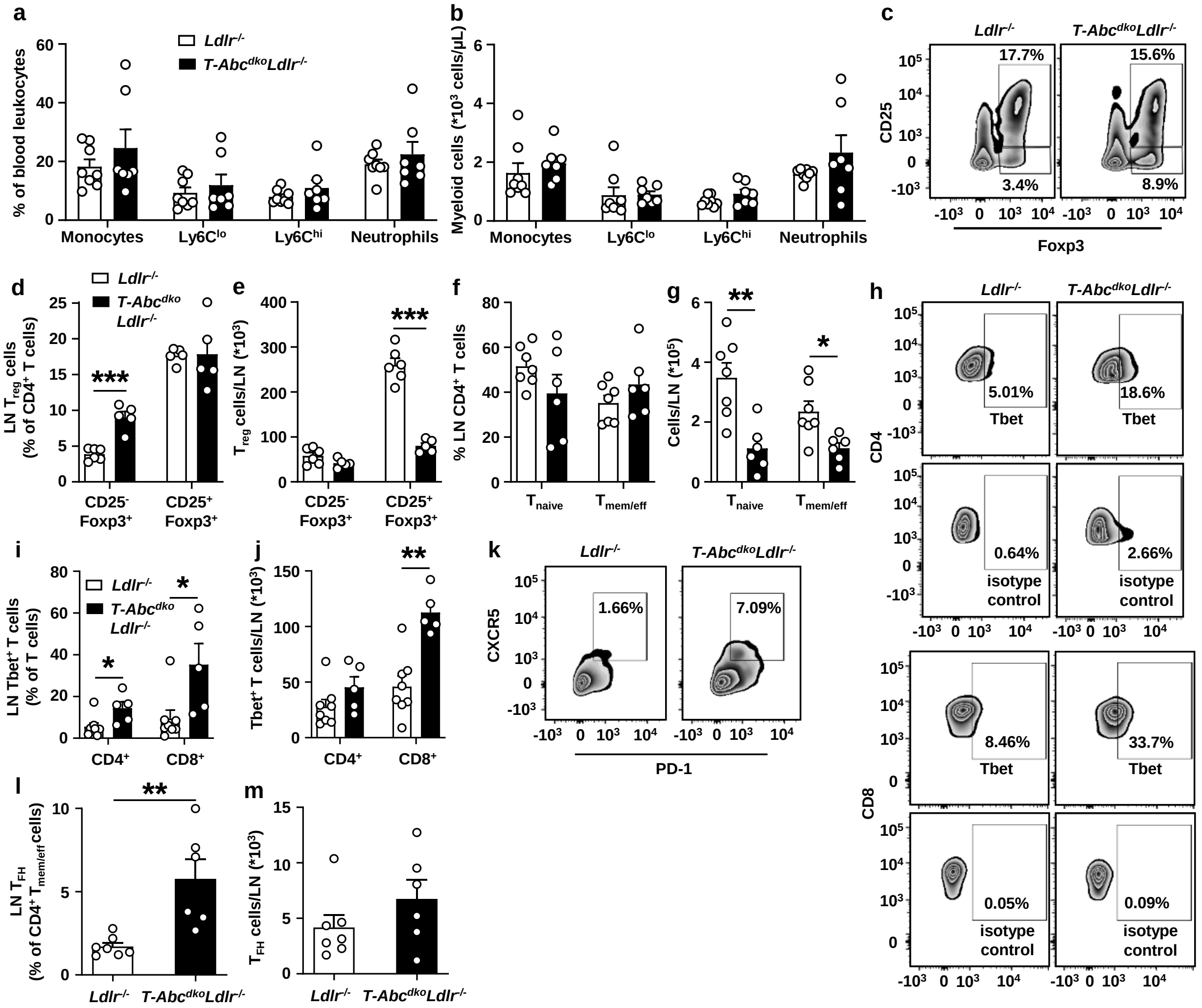

Supplementary Fig. 24 Effects of T cell *Abca1/Abcg1* deficiency on blood myeloid cells and lymph node T cell subsets in middle-aged *Ldlr*$^{-/-}$ mice. *Ldlr*$^{-/-}$ and *T-Abc*^{dko}*Ldlr*$^{-/-}$ mice were fed a chow diet for 12-13 months. Blood and para-aortic LNs were collected, cells were stained with the indicated antibodies, and analyzed by flow cytometry. (**a-b**) Monocytes, Ly6C^{lo} and Ly6C^{hi} monocyte subsets, and neutrophils as a percentage of total leukocytes (**a**), and (**b**) expressed as concentration in blood after correction for total blood leukocyte counts. n=8 *Ldlr*$^{-/-}$ and n=7 *T-Abc*^{dko}*Ldlr*$^{-/-}$ mice. (**c-e**) Representative flow cytometry plots of (**c**) CD25$^-$Foxp3$^+$ and CD25$^+$Foxp3$^+$ T$_{reg}$ cells gated as in Supplementary Fig. 18a, (**d**) CD25$^-$Foxp3$^+$ (*p*=0.00012) and CD25$^+$Foxp3$^+$ T$_{reg}$ cells as a percentage of para-aortic LN CD4$^+$ T cells after correction for isotype control, and (**e**) CD25$^-$Foxp3$^+$ and CD25$^+$Foxp3$^+$ T$_{reg}$ cells (*p*=0.000003) expressed as cells/LN after correction for total para-aortic LN cell number. n=6 *Ldlr*$^{-/-}$ and n=5 *T-Abc*^{dko}*Ldlr*$^{-/-}$ mice. (**f-g**) CD4$^+$ T$_{naive}$ and T$_{mem/eff}$ cells as a percentage of CD4$^+$ T cells in para-aortic LNs (**f**) and (**g**) CD4$^+$ T$_{naive}$ (*p*=0.0028) and T$_{mem/eff}$ (*p*=0.0136) cells expressed as cells/LN after correction for total para-aortic LN cell number. n=7 *Ldlr*$^{-/-}$ and n=6 *T-Abc*^{dko}*Ldlr*$^{-/-}$ mice. (**h-j**) Representative flow cytometry plots of (**h**) CD4$^+$Tbet$^+$ and CD8$^+$Tbet$^+$ cells gated as in Supplementary Fig. 18e, (**i**) CD4$^+$Tbet$^+$ (*p*=0.022) and CD8$^+$Tbet$^+$ (*p*=0.0185) cells as percentages of CD4$^+$ and CD8$^+$ para-aortic LN cells after correction for isotype control, and (**j**) CD4$^+$Tbet$^+$ and CD8$^+$Tbet$^+$ (*p*=0.0019) cells expressed as cells/LN after correction for total para-aortic LN cell number. n=8 *Ldlr*$^{-/-}$ and n=5 *T-Abc*^{dko}*Ldlr*$^{-/-}$ mice. (**k-m**) Representative flow cytometry plots of (**k**) CD4$^+$CD44$^+$CD62L$^-$CXCR5$^+$PD-1$^+$ T$_{FH}$ cells

gated as in Supplementary Fig. 18d, (**l**) T_{FH} cells (p=0.0038) as a percentage of $CD4^+$ $T_{mem/eff}$ cells, and (**m**) expressed as cells/LN. n=7 *Ldlr*$^{-/-}$ and n=6 *T-Abc*^{dko}*Ldlr*$^{-/-}$ mice. For all panels, error bars represent SEM, and biologically independent samples were included. *p* value was determined by unpaired two-tailed Student's t-test. **p*<0.05, ***p*<0.01, ****p*<0.001. Source data are provided as a Source Data file.

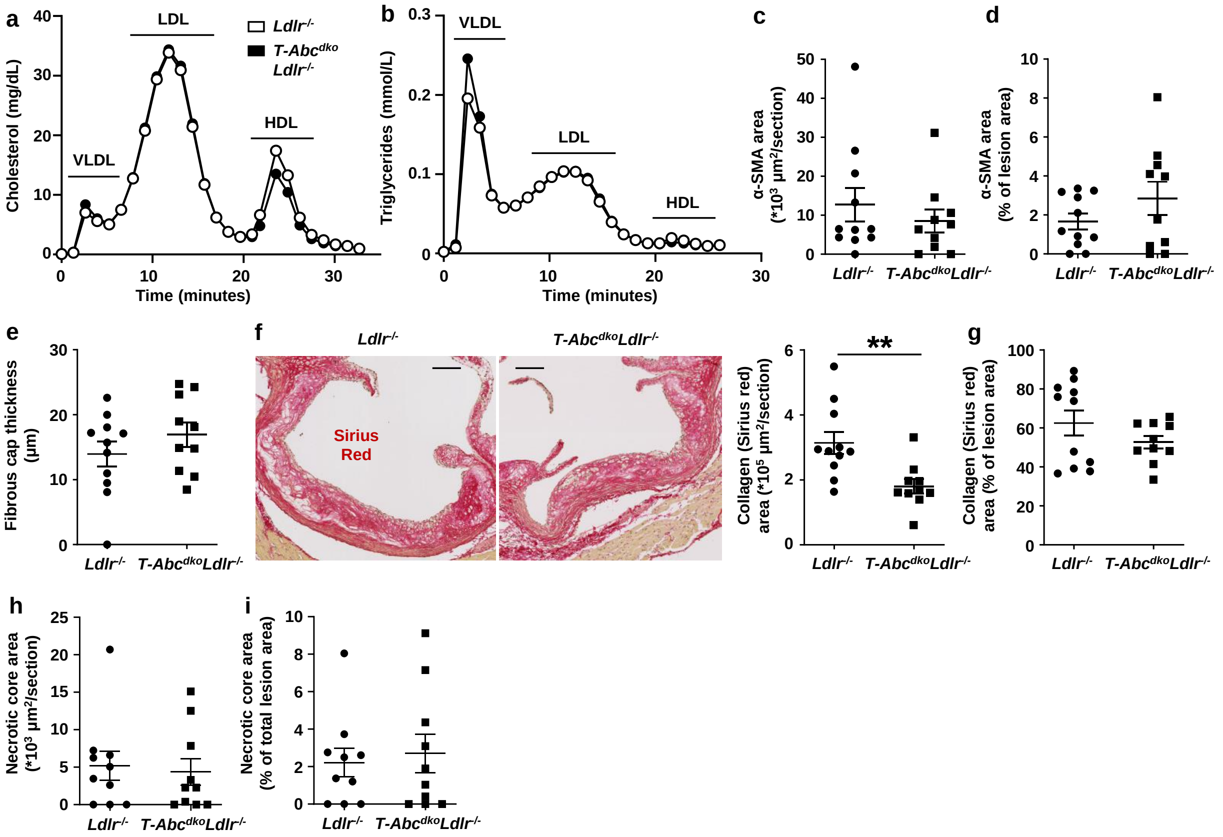

Supplementary Fig. 25 Effects of T cell *Abca1/Abcg1* deficiency on plasma lipids and plaque stability in middle-aged *Ldlr*$^{-/-}$ mice. *Ldlr*$^{-/-}$ and *T-Abc*^{dko}*Ldlr*$^{-/-}$ mice were fed a chow diet for 12-13 months. (**a-b**) Lipoproteins from pooled plasma samples (n=11 *Ldlr*$^{-/-}$ and n=10 *T-Abc*^{dko}*Ldlr*$^{-/-}$ mice per pool) were separated using FPLC and total cholesterol (**a**) and triglyceride (**b**) levels were determined. (**c-i**) Hearts were isolated, sectioned, and paraffin sections of the aortic root were stained for (**c-d**) α-SMA, (**e-g**) Sirius Red, or (**h-i**) H&E. (**c-d**) Total α-SMA^+ area (**c**) and α-SMA^+ area as a percentage of total atherosclerotic lesion area (**d**). n=11 *Ldlr*$^{-/-}$ and n=10 *T-Abc*^{dko}*Ldlr*$^{-/-}$ mice. (**e**) Fibrous cap thickness. n=11 *Ldlr*$^{-/-}$ and n=10 *T-Abc*^{dko}*Ldlr*$^{-/-}$ mice. (**f**) Representative pictures of Sirius Red staining (left) and quantification (right) of total collagen (Sirius Red^+) area (p=0.0043). Scale bar represents 100 µm. (**g**) Collagen area as a percentage of total atherosclerotic lesion area. (**f-g**) n=11 *Ldlr*$^{-/-}$ and n=10 *T-Abc*^{dko}*Ldlr*$^{-/-}$ mice. (**h-i**) Total necrotic core area (**i**) and necrotic core area as a percentage of total atherosclerotic lesion area (**j**). n=10 *Ldlr*$^{-/-}$ and n=10 *T-Abc*^{dko}*Ldlr*$^{-/-}$ mice. For (**c-i**), error bars represent SEM, and biologically independent samples were included. *p* value was determined by unpaired two-tailed Student's t-test. ***p*<0.01. Source data are provided as a Source Data file.

Supplementary Table 1. Plasma cholesterol and triglyceride levels in young wild-type mice, aged wild-type mice, *Ldlr*$^{-/-}$, and *T-Abc*^{dko}*Ldlr*$^{-/-}$ mice fed chow diet or Western-type diet (WTD).

Genotype	Diet	Plasma cholesterol (mg/dL)	Plasma triglycerides (mmol/L)
young wild-type	Chow diet (3 months)	99.8 ± 5.6	1.42 ± 0.11
aged wild-type	Chow diet (24 months)	83.6 ± 8.1	0.83*** ± 0.06
Ldlr$^{-/-}$	WTD	919.9 ± 52.4	1.90 ± 0.14
T-Abc^{dko}*Ldlr*$^{-/-}$	WTD	780.2* ± 33.1	2.13 ± 0.19
Ldlr$^{-/-}$	Chow diet (28 weeks)	283.8 ± 15.1	1.72 ± 0.17
T-Abc^{dko}*Ldlr*$^{-/-}$	Chow diet (28 weeks)	295.3 ± 16.2	1.60 ± 0.16
Ldlr$^{-/-}$	Chow diet (12-13 months)	255.8 ± 14.8	1.38 ± 0.22
T-Abc^{dko}*Ldlr*$^{-/-}$	Chow diet (12-13 months)	249.1 ± 18.6	1.44 ± 0.09

Plasma cholesterol and triglyceride levels were determined using enzymatic kits. n=16 young and n=16 aged wild-type mice on chow diet. n=15 *Ldlr*$^{-/-}$ and n=15 *T-Abc*^{dko}*Ldlr*$^{-/-}$ mice on WTD. n=18 *Ldlr*$^{-/-}$ and n=16 *T-Abc*^{dko}*Ldlr*$^{-/-}$ mice on chow diet for 28 weeks. n=11 *Ldlr*$^{-/-}$ and n=10 *T-Abc*^{dko}*Ldlr*$^{-/-}$ mice on chow diet for 12-13 months. For all mouse groups, ± SEM is shown, and biologically independent samples were included. *p* value was determined by unpaired two-tailed Student's t-test. *$p<0.05$, ***$p<0.001$. Source data are provided as a Source Data file.

Supplementary Table 2. Summary of effects of T cell *Abca1/Abcg1* deficiency on T cell subsets in para-aortic LNs from *Ldlr*$^{-/-}$ mice fed chow diet.

T cell subset	Changes in percentages of para-aortic LN cells		Changes in cells/para-aortic LN
	Cell type %	Change	
$CD25^+Foxp3^+$ T_{reg} cells	$CD4^+$ T cells	↔	~75% ↓
$CD25^-Foxp3^+$ T_{reg} cells	$CD4^+$ T cells	~2.5-fold ↑	↔
T_{FH}	$CD4^+$ $T_{mem/eff}$ cells	~1.7-fold ↑	↔
$CD4^+Tbet^+$	$CD4^+$ T cells	~11-fold ↑	↔
$CD8^+Tbet^+$	$CD8^+$ T cells	~6.7-fold ↑	~2-fold ↑

Para-aortic LNs were isolated and total LN cells were stained with the indicated antibodies and analyzed by flow cytometry. Data are shown in Figure 7.

Supplementary Table 3. Reagents and resources.

REAGENT or RESOURCE	SOURCE	IDENTIFIER
Antibodies		
Rat anti-mouse CD115-PE	Biolegend	Cat# 135506; RRID:AB_1937253; clone AFS98; 1/200 dilution
Rat anti-mouse Ly6-C/G-PerCP-Cy5.5	BD Biosciences	Cat# 561103; RRID:AB_10562568; clone RB6-8C5; 1/200 dilution
Rat anti-mouse CD45-APC-Cy7	BD Biosciences	Cat# 557659; RRID:AB_396774; clone 30-F11; 1/200 dilution
Rat anti-mouse CD25-PECy7	eBioscience	Cat# 25-0251-82; RRID:AB_469608; clone PC61.5; 1:200 dilution
Rat anti-mouse CD4-PB	Biolegend	Cat# 100427; RRID:AB_493646; clone GK1.5; 1/200 dilution
Rat anti-mouse CD4-APC	eBioscience	Cat# 17-0041-82; RRID:AB_469320; clone GK1.5; 1/200 dilution
Rat anti-mouse CD4-APC-Cy7	Biolegend	Cat# 100413; RRID:AB_312698; clone GK1.5; 1/200 dilution
Rat anti-mouse CD4-PE	Biolegend	Cat# 100407; RRID:AB_312692; clone GK1.5; 1/200 dilution
Rat anti-mouse CD4-FITC	eBioscience	Cat# 11-0042-82; RRID:AB_464896; clone RM4-5; 1/200 dilution
Rat anti-mouse CD8-PB (eFluor 450)	eBioscience	Cat# 48-0083-82; RRID:AB_11218504; clone eBioH35-17.2 (H35-17.2); 1/200 dilution
Rat anti-mouse CD8-FITC	eBioscience	Cat# 11-0083-82; RRID:AB_657764; clone eBioH35-17.2 (H35-17.2); 1/200 dilution
Rat anti-mouse CD8-PE	eBioscience	Cat# 12-0083-82; RRID:AB_657767; clone eBioH35-17.2 (H35-17.2); 1/200 dilution
Rat anti-mouse CD8-APC	Biolegend	Cat# 126613; RRID:AB_657767; clone YTS156.7.7; 1/200 dilution
Armenian hamster anti-mouse TCRβ-PB	Biolegend	Cat# 109226; RRID:AB_1027649; clone H57-597; 1/200 dilution
Armenian hamster anti-mouse TCRβ-APC	Biolegend	Cat# 109211; RRID:AB_313434; clone H57-597; 1/200 dilution

Armenian hamster anti-mouse TCRβ-PerCP-Cy5.5	Biolegend	Cat# 109227; RRID:AB_1575176; clone H57-597; 1/200 dilution
Armenian hamster anti-mouse PD1-PB (eFluor 450)	eBioscience	Cat# 48-9985; RRID:AB_2574138; clone J43; 1/200 dilution
Rat anti-mouse PD1-PE	Biolegend	Cat# 109103; RRID:AB_313420; clone RMP1-30; 1/200 dilution
Mouse anti-mouse Tbet-PE	eBioscience	Cat# 12-5825; RRID:AB_925761; clone eBio4B10 (4B10); 1/100 dilution
Mouse IgG1 kappa isotype control-PE (for Tbet- and Eomes-PE)	eBioscience	Cat# 12-4714-42; RRID:AB_470060; clone P3.6.2.8.1; 1/100 dilution
Mouse anti-mouse Tbet-AF488	eBioscience	Cat# 53-5825-80; RRID:AB_2815215; clone eBio4B10 (4B10); 1/100 dilution
Mouse IgG1 kappa isotype control-AF488 (for Tbet-AF488)	eBioscience	Cat# 53-4714-80; RRID:AB_470230; clone P3.6.2.8.1; 1/100 dilution
Rat anti-mouse Eomes-PE	eBioscience	Cat# 12-4875-82; RRID:AB_1603275; clone Dan11mag; 1/100 dilution
Rat anti-mouse CXCR5-FITC	Biolegend	Cat# 145519; RRID:AB_2562865; clone L138D7; 1/400 dilution
Rat anti-mouse CD44-PB (eFluor 450)	eBioscience	Cat# 48-0441-82; RRID:AB_1272246; clone IM7; 1/200 dilution
Rat anti-mouse CD44-PE-Cy7	eBioscience	Cat# 25-0441-82; RRID:AB_469623; clone IM7; 1/200 dilution
Rat anti-mouse CD62L-APC	eBioscience	Cat# 17-0621-82; RRID:AB_469410; clone MEL-14; 1/200 dilution
Rat anti-mouse CD62L-FITC	Biolegend	Cat# 104405; RRID:AB_313092; clone MEL-14; 1/200 dilution
Rat anti-mouse CD62L-PB	Biolegend	Cat# 104423; RRID:AB_493381; clone MEL-14; 1/200 dilution
Rat anti-mouse Foxp3-APC	eBioscience	Cat# 17-5773-80; RRID:AB_469456; clone FJK-16s; 1/100 dilution
Rat IgG2a kappa isotype control-APC (for Foxp3-APC)	eBioscience	Cat# 17-4321-81; RRID:AB_470181; clone eBR2a; 1/100 dilution
Mouse anti-mouse Fas-AF488	eBioscience	Cat# 53-0951-82; RRID:AB_10671269; clone 15A7; 1/200 dilution

Mouse anti-mouse Bcl2-FITC	eBioscience	Cat# 11-6992-42; RRID:AB_10734060; clone 10C4; 1/40 dilution
Mouse IgG1 kappa isotype control-FITC (for Bcl2-FITC)	eBioscience	Cat# 11-4714-82; RRID:AB_470022; 1/40 dilution
Armenian hamster anti-mouse CTLA4-Brilliant Violet 421 (PB)	Biolegend	Cat# 106311; RRID:AB_10901170; clone UC10-4B9; 1/200 dilution
Rat anti-mouse TIM3-APC	Biolegend	Cat# 134007; RRID:AB_2562997; clone B8.2C12; 1/200 dilution
Rat anti-mouse LAG3-APC	Biolegend	Cat# 125209; RRID:AB_10639935; clone C9B7W; 1/200 dilution
Rat anti-mouse CD24-PE-Cy7	Biolegend	Cat# 101821; RRID:AB_756047; clone<br>M1/69; 1/200 dilution
Rat anti-mouse CD69-APC	Biolegend	Cat# 104513; RRID:AB_492844; clone H1.2F3; 1/200 dilution
Rat anti-mouse Granzyme B-FITC	eBioscience	Cat# 11-8898-80; RRID:AB_10732989; clone NGZB; 1/100 dilution
Rat IgG2a kappa Isotype Control-FITC (for Granzyme B-FITC)	eBioscience	Cat# 11-4321-80; RRID:AB_1834375; 1/100 dilution
Rat anti-mouse IFN-γ-FITC	eBioscience	Cat# 11-7311-81; RRID:AB_465411; clone XMG1.2; 1/100 dilution
Rat anti-mouse LAMP-1 (CD107a)	Biolegend	Cat# 121605; RRID:AB_572006; clone 1D4B; 1/200 dilution
Rabbit anti-mouse SAPK/JNK (for Western blot)	Cell Signaling Technology	Cat# 9252; RRID:AB_2250373; 1/1000 dilution
Mouse anti-mouse Phospho-SAPK/JNK (Thr183/Tyr185) (for Western blot)	Cell Signaling Technology	Cat# 9255; RRID:AB_2307321; 1/1000 dilution
Rabbit anti-mouse HSP90 (for Western blot)	Cell Signaling Technology	Cat# 4874; RRID:AB_2121214; 1/1000 dilution
Rat anti-mouse gasdermin D (for Western blot)	Genentech [1]	N/A; clone 17G2G9; 1/1000 dilution
Goat anti-Mouse IgG (H+L) Secondary Antibody, HRP (for Western blot; for phospho-SAPK/JNK)	Invitrogen	Cat# 31430; RRID:AB_228307; 1/2000 dilution
Goat anti-Rabbit IgG (H+L) Secondary Antibody, HRP (for Western blot; for SAPK/JNK and HSP90)	Invitrogen	Cat# 31460; RRID:AB_228341; 1/2000 dilution
Goat anti-rat IgG, HRP-linked (for Western blot; for gasdermin D)	Cell Signaling Technology	Cat# 7077; AB_10694715; 1/1000 dilution
Rabbit anti-mouse Actin, Smooth Muscle (for histology)	Lab Vision	Cat# Epredia RB-9010-P; RRID:AB_149757; 1/200 dilution
Rabbit anti-human CD3 (for histology)	Dako	Cat# A0452; RRID:AB_2335677; 1/250 dilution

Goat anti-rabbit IgG antibody (H+L), biotinylated (for SMA and CD3 stainings)	Vector Laboratories	Cat# BA-1000; AB_2313606; 1/250 dilution
Rat Anti-Mouse/Human Mac-2 (Galectin-3) (for histology)	Cedarlane	Cat# CL8942AP; RRID:AB_2534074;clone M3/38; 1/10000 dilution
Goat anti-Rat IgG (H+L) Cross-Adsorbed Secondary Antibody, AF488 (for Mac-2 staining)	Invitrogen	Cat# A-11006; RRID:AB_10060357; 1/200 dilution
Goat anti-rat IgG antibody (H+L), biotinylated (for Mac-2 staining)	Vector Laboratories	Cat# BA-9400; AB_ 2336202; 1/125 dilution
LEAF Purified rat anti-mouse CD3 (for stimulation)	Biolegend	Cat# 100208; RRID:AB_312665; clone 17A2; amount 5µg/mL
Chemicals and Recombinant Proteins		
Incucyte Caspase3/7 Red Reagent	Sartorius	Cat# 4704
Cell Proliferation Dye eFluor 450 (CFSE-PB)	eBioscience	Cat# 65-0842-85
Filipin III	Sigma-Aldrich	Cat# F4767
Filipin complex	Sigma-Aldrich	Cat# F9765
Choleratoxin B-FITC	Sigma-Aldrich	Cat# C1655
MitoSOX Red Mitochondrial Superoxide Indicator	Invitrogen	Cat# M36008
Mitotracker Green FM	Invitrogen	Cat# M7514
MitoTracker Red CMXRos	Invitrogen	Cat# M7512
Bodipy 493/503	Invitrogen	Cat# D3922
Bodipy C10	Ulf Diederichsen Lab, Georg-August-Universität Göttingen, Germany [2]	N/A
VECTASHIELD Antifade Mounting Medium with DAPI	Vector Laboratories	Cat# H-1200
VECTASHIELD Antifade Mounting Medium	Vector Laboratories	Cat# H-1000
ProLong Gold Antifade Mountant with DAPI	Invitrogen	Cat# P36935
Hoechst 33342	Invitrogen	Cat# H3570
Oil Red O	Sigma-Aldrich	Cat# O0625
Sirius Red (Direct Red 80)	Sigma-Aldrich	Cat# 365548
Recombinant murine IL-2	Peprotech	Cat# 212-12
Animal-Free Recombinant Human TGF-β1 (CHO derived)	Peprotech	Cat# AF-100-21C
Recombinant murine IFN-γ	Peprotech	Cat# 315-05
Reconstituted HDL	Meridian Bioscience	Cat# A34275H
Liberase TM Research Grade	Roche	Cat# 5401127001
Liberase TH Research Grade	Roche	Cat# 5401135001
Deoxyribonuclease I from bovine pancreas	Sigma-Aldrich	Cat# DN25
Hyaluronidase from bovine testes	Sigma-Aldrich	Cat# H3506

Gentamicin	Gibco	Cat# 12664735
Proteinase K	Merck	Cat# 1.24568.0100
Perm/Wash Buffer	BD Biosciences	Cat# 554723
Vectastain ABC-HRP Kit, peroxidase (for SMA and Mac-2 stainings)	Vector Laboratories	Cat# PK-4000
Vectastain Elite ABC Kit (for CD3 staining)	Vector Laboratories	Cat# PK-6100
Cholesterol reagent	Roche	Cat# 11489232
Cholesterol Standard FS	Diasys Diagnostic Systems	Cat# 113009910026
Triglyceride FS	Diasys Diagnostic Systems	Cat# 157109910917
Precimat Glycerol	Roche	Cat# 10166588130
BD Pharm Lyse	BD Biosciences	Cat# 555899
Critical Commercial Assays		
Dynabeads Mouse T-Activator CD3/CD28 for T-Cell Expansion and Activation	Gibco	Cat# 11452D
Dead Cell Removal Kit	Miltenyi Biotec	Cat# 130-090-101
Pan T Cell Isolation Kit II, mouse	Miltenyi Biotec	Cat# 130-095-130
CD4 (L3T4) MicroBeads, mouse	Miltenyi Biotec	Cat# 130-117-043
CD8a (Ly-2) MicroBeads, mouse	Miltenyi Biotec	Cat# 130-117-044
Mouse Regulatory T Cell Staining Kit #1	eBioscience	Cat# 88-8111
Foxp3/Transcription Factor Staining Buffer Set	eBioscience	Cat# 00-5523
In Situ Cell Death Detection Kit, TMR red	Roche	Cat# 12156792910
Mouse IFN-gamma Quantikine ELISA Kit	R&D Systems	Cat# MIF00
CyQUANT LDH Cytotoxicity Assay	Invitrogen	Cat# C20300
Experimental Models: Organisms/Strains		
Mouse: Abca1$^{fl/fl}$Abcg1$^{fl/fl}$: B6.Cg-Abca1^{tm1Jp} Abcg1^{tm1Tall}/J	The Jackson Laboratory	Cat# JAX:021067; RRID:IMSR_JAX:021067
Mouse: CD4Cre: Tg(Cd4-cre)1Cwi/BfluJ	The Jackson Laboratory	Cat# JAX:017336; RRID:IMSR_JAX:017336
Mouse: Ldlr$^{-/-}$: B6.129S7-Ldlr^{tm1Her}/J	The Jackson Laboratory	Cat# JAX:002207; RRID:IMSR_JAX:002207
Mouse: wild-type: C57BL/6J	The Jackson Laboratory	Cat# JAX:000664; RRID:IMSR_JAX:000664
Animal diets		
Chow diet (10% fat, 23% protein, 67% carbohydrates)	Ssniff Spezialdiäten GmbH	Cat# V1554-703
Western-type diet (40% fat, 0.15% cholesterol)	Research Diets	Cat# D12079B
Bacterial strains		

E. coli K-12 MG1655	Marjon G.J. de Vos Lab, University of Groningen, the Netherlands	N/A
Oligonucleotides		
Mouse *Abca1fl/fl* forward primer 5'-GTGAATGGGCAATTCGCAAACT-3'	[3]	N/A
Mouse *Abca1fl/fl* reverse primer 5'-AGATCTCCCCTCCTTGACAATGC-3'	[3]	N/A
Mouse *Abcg1fl/fl* forward primer 5'-TGTTCAGGAGGCCATGATGGT-3'	[3]	N/A
Mouse *Abcg1fl/fl* reverse primer 5'-TGGCCAGGCGTTTCCG-3' T	[3]	N/A
Mouse *Cdkn1a* forward primer 5'-TTGCCAGCAGAATAAAAGGTG-3'	[4]	N/A
Mouse *Cdkn1a* reverse primer 5'-TTTGCTCCTGTGCGGAAC-3'	[4]	N/A
Mouse *Tnf* forward primer 5'-GTAGCCCACGTCGTAGCAAAC-3'	[5]	N/A
Mouse *Tnf* reverse primer 5'-AGTTGGTTGTCTTTGAGATCCATG-3'	[5]	N/A
Mouse *Foxp3* forward primer 5'-TCTTGCCAAGCTGGAAGACT-3'	[6]	N/A
Mouse *Foxp3* reverse primer 5'-GGGGTTCAAGGAAGAAGAGG-3'	[6]	N/A
Mouse *Il10* forward primer 5'-GCTCTTACTGACTGGCATGAG-3'	[7]	N/A
Mouse *Il10* reverse primer 5'-CGCAGCTCTAGGAGCATGTG-3'	[7]	N/A
Mouse *Tgfb1* forward primer 5'-GCCCTTCCTGCTCCTCATG-3'	[8]	N/A
Mouse *Tgfb1* reverse primer 5'-CCGCACACAGCAGTTCTTCTC-3'	[8]	N/A
Mouse *Bcl2* forward primer 5'-GAACTGGGGGAGGATTGTGG-3'	[9]	N/A
Mouse *Bcl2* reverse primer 5'-ACCTACCCAGCCTCCGTTAT-3'	[9]	N/A
Mouse *Itgam* forward primer 5'-TCAGAGAATGTCCTCAGCAG-3'	[5]	N/A
Mouse *Itgam* reverse primer 5'-TGAGACAAACTCCTTCATCTTC-3'	[5]	N/A
Mouse *Ccl2* forward primer 5'-GCTGGAGAGCTACAAGAGGATCA-3'	[8]	N/A
Mouse *Ccl2* reverse primer 5'-ACAGACCTCTCTCTTGAGCTTGGT-3'	[8]	N/A

Mouse *Il6* forward primer 5’-CTGCAAGAGACTTCCATCCAGTT-3’	[10]	N/A
Mouse *Il6* reverse primer 5’-AGGGAAGGCCGTGGTTGT-3’	[10]	N/A
Mouse *Il1b* forward primer 5’-TGCAGCTGGAGAGTGTGG-3’	[5]	N/A
Mouse *Il1b* reverse primer 5’-TGCTTGTGAGGTGCTGATG-3’	[5]	N/A
Mouse *Ldlr* forward primer 5’-CATATGCATCCCCAGTCTTTG-3’	[11]	N/A
Mouse *Ldlr* reverse primer 5’-GCAGTGCTCCTCATCTGACTTG-3’	[11]	N/A
Mouse *Hmgcr* forward primer 5’-AGCTTGCCCGAATTGTATGTG-3’	[11]	N/A
Mouse *Hmgcr* reverse primer 5’-TCTGTTGTGAACCATGTGACTTC-3’	[11]	N/A
Mouse *Hmgcs* forward primer 5’-GCCGTGAACTGGGTCGAA-3’	[12]	N/A
Mouse *Hmgcs* reverse primer 5’-GCATATATAGCAATGTCTCCTGCAA-3’	[12]	N/A
Mouse *Srebf2* forward primer 5’-CGACGAGATGCTACAGTTTG-3’	[11]	N/A
Mouse *Srebf2* reverse primer 5’-GGTAGGAGAGACTTTGACCTG-3’	[11]	N/A
Mouse *Srebf1* forward primer 5’-TTACTCGAGCCTGCCTTCAG-3’	This paper	N/A
Mouse *Srebf1* reverse primer 5’-TAGATGGTGGCTGCTGAGTG-3’	This paper	N/A
Mouse *Acat1* forward primer 5’-GAAGGCTCACTCATTTGTCAGA-3’	[13]	N/A
Mouse *Acat1* reverse primer 5’-GTCTCGGTAAATAAGTGTAGGCG-3’	[13]	N/A
Mouse *Cdkn2a* forward primer 5’-AATCTCCGCGAGGAAAGC-3’	[4]	N/A
Mouse *Cdkn2a* reverse primer 5’-GTCTGCAGCGGACTCCAT-3’	[4]	N/A
Mouse *Il23a* forward primer 5’-ATGCTGGATTGCAGAGCAGTA-3’	[14]	N/A
Mouse *Il23a* reverse primer 5’-ACGGGGCACATTATTTTTAGTCT-3’	[14]	N/A
Mouse *Icam1* forward primer 5’-ACTGCACGTGCTGTATGGTC-3’	This paper	N/A

Mouse *Icam1* reverse primer 5'-CTGCAGGTCATCTTAGGAGATG-3'	This paper	N/A
Mouse *Retnla* forward primer 5'-TCCCAGTGAATACTGATGAGA-3'	[15]	N/A
Mouse *Retnla* reverse primer 5'-CCACTCTGGATCTCCCAAGA-3'	[15]	N/A
Mouse *Chil3* forward primer 5'-AGAAGGGAGTTTCAAACCTGGT-3'	[16]	N/A
Mouse *Chil3* reverse primer 5'-GTCTTGCTCATGTGTGTAAGTGA-3'	[16]	N/A
Software and Algorithms		
FACS DiVa software V8.0.3	BD Biosciences	https://www.bdbiosciences.com/us/instruments/research/software/flow-cytometry-acquisition/bdfacsdiva-software/m/111112/overview
FlowJo version 10.6.2	FlowJo	https://www.flowjo.com/solutions/flowjo/downloads
GraphPad Prism version 9	GraphPad Software	https://www.graphpad.com/scientificsoftware/prism/
ImageJ 1.51k_Java 1.6.0_24	NIH	https://imagej.nih.gov/ij/index.html
IncuCyte ZOOM 2018A	Sartorius	https://www.essenbioscience.com/en/resources/incucyte-zoom-resources-support/software-modules-incucyte-zoom/
SymPhoTime 64 version 2.6	PicoQuant	https://www.picoquant.com/products/category/software/symphotime-64-fluorescence-lifetime-imaging-and-correlation-software
FLIMfit 5.1.1	FLIMfit, Open Microscopy Environment	http://flimfit.org/downloads/
Zen 3.4	Zeiss	https://www.zeiss.com/microscopy/us/products/microscope-software/zen-lite.html
ChromNAV version 1.0	Jasco	https://jasco-chromnav.software.informer.com/1.0/

REFERENCES

1. Aglietti, R. A. *et al.* GsdmD p30 elicited by caspase-11 during pyroptosis forms pores in membranes. *Proc Natl Acad Sci U S A* **113**, 7858–63 (2016).

2. Vyšniauskas, A., Qurashi, M. & Kuimova, M. K. A Molecular Rotor that Measures Dynamic Changes of Lipid Bilayer Viscosity Caused by Oxidative Stress. *Chemistry - A European Journal* **22**, 13210–13217 (2016).

3. Westerterp, M. *et al.* Cholesterol Accumulation in Dendritic Cells Links the Inflammasome to Acquired Immunity. *Cell Metab* **65**, 3176–3185 (2017).

4. Demaria, M. *et al.* An essential role for senescent cells in optimal wound healing through secretion of PDGF-AA. *Developmental Cell* **31**, 722–33 (2014).

5. van der Heijden, R. A. *et al.* High-fat diet induced obesity primes inflammation in adipose tissue prior to liver in C57BL/6j mice. *Aging* **7**, 256–68 (2015).

6. Subramanian, M., Thorp, E. & Tabas, I. Identification of a non-growth factor role for GM-CSF in advanced atherosclerosis: Promotion of macrophage apoptosis and plaque necrosis through IL-23 signaling. *Circulation Research* **116**, e13-24 (2015).

7. Jin, W. *et al.* Stromal Cell-Derived Factor-1 Enhances the Therapeutic Effects of Human Endometrial Regenerative Cells in a Mouse Sepsis Model. *Stem Cells International* **2020**, 4820543 (2020).

8. Rensen, S. S. *et al.* Neutrophil-Derived Myeloperoxidase Aggravates Non-Alcoholic Steatohepatitis in Low-Density Lipoprotein Receptor-Deficient Mice. *PLoS ONE* **7**, e52411 (2012).

9. Sun, T. C. *et al.* Melatonin Inhibits Oxidative Stress and Apoptosis in Cryopreserved Ovarian Tissues via Nrf2/HO-1 Signaling Pathway. *Frontiers in Molecular Biosciences* **7**, 163 (2020).

10. Ding, S. *et al.* Resveratrol reduces the inflammatory response in adipose tissue and improves adipose insulin signaling in high-fat diet-fed mice. *PeerJ* **2018**, e5173 (2018).

11. Bartuzi, P. *et al.* CCC- and WASH-mediated endosomal sorting of LDLR is required for normal clearance of circulating LDL. *Nature Communications* **7**, 10961 (2016).

12. Armstrong, A. J., Gebre, A. K., Parks, J. S. & Hedrick, C. C. ATP-Binding Cassette Transporter G1 Negatively Regulates Thymocyte and Peripheral Lymphocyte Proliferation. *The Journal of Immunology* **184**, 173–183 (2010).

13. Yao, Y. *et al.* Identification of Caspase-6 as a new regulator of alternatively activated macrophages. *Journal of Biological Chemistry* **291**, 17450–66 (2016).

14. Du, Y. N. *et al.* SGK1-FoxO1 signaling pathway mediates Th17/Treg imbalance and target organ inflammation in angiotensin II-induced hypertension. *Frontiers in Physiology* **9**, 1581 (2018).

15. dos Santos, A. G. *et al.* Trichoderma asperelloides Spores Downregulate dectin1/2 and TLR2 Receptors of Mice Macrophages and Decrease Candida parapsilosis Phagocytosis Independent of the M1/M2 Polarization. *Frontiers in Microbiology* **8**, 1681 (2017).

16. Dong, J. *et al.* Quercetin reduces obesity-associated ATM infiltration and inflammation in mice: A mechanism including AMPKα1/SIRT. *Journal of Lipid Research* **55**, 363–74 (2014).
