# Supplementary material for: T cell cholesterol efflux suppresses apoptosis and senescence and increases atherosclerosis in middle aged mice
Source: Nat Commun. 2022 Jul 1;13:3799. doi: 10.1038/s41467-022-31135-4 (PMC9249754; doi:10.1038/s41467-022-31135-4)
Supplement: Supplementary file 3 — Reporting Summary [file 41467_2022_31135_MOESM3_ESM.pdf]

nature portfolio

Corresponding author(s): Marit Westerterp

Last updated by author(s): Apr 10, 2022

Reporting Summary

Nature Portfolio wishes to improve the reproducibility of the work that we publish. This form provides structure for consistency and transparency in reporting. For further information on Nature Portfolio policies, see our Editorial Policies and the Editorial Policy Checklist.

Please do not complete any field with "not applicable" or n/a. Refer to the help text for what text to use if an item is not relevant to your study.
For final submission: please carefully check your responses for accuracy; you will not be able to make changes later.

Statistics

For all statistical analyses, confirm that the following items are present in the figure legend, table legend, main text, or Methods section.

n/a	Confirmed	
☐	☒	The exact sample size (*n*) for each experimental group/condition, given as a discrete number and unit of measurement
☐	☒	A statement on whether measurements were taken from distinct samples or whether the same sample was measured repeatedly
☐	☒	The statistical test(s) used AND whether they are one- or two-sided<br>*Only common tests should be described solely by name; describe more complex techniques in the Methods section.*
☒	☐	A description of all covariates tested
☒	☐	A description of any assumptions or corrections, such as tests of normality and adjustment for multiple comparisons
☐	☒	A full description of the statistical parameters including central tendency (e.g. means) or other basic estimates (e.g. regression coefficient) AND variation (e.g. standard deviation) or associated estimates of uncertainty (e.g. confidence intervals)
☐	☒	For null hypothesis testing, the test statistic (e.g. *F*, *t*, *r*) with confidence intervals, effect sizes, degrees of freedom and *P* value noted<br>*Give P values as exact values whenever suitable.*
☒	☐	For Bayesian analysis, information on the choice of priors and Markov chain Monte Carlo settings
☒	☐	For hierarchical and complex designs, identification of the appropriate level for tests and full reporting of outcomes
☒	☐	Estimates of effect sizes (e.g. Cohen's *d*, Pearson's *r*), indicating how they were calculated

Our web collection on statistics for biologists contains articles on many of the points above.

Software and code

Policy information about availability of computer code

Data collection	FACS DiVa software V8.0.3 (BD Bioscience; for flow cytometry experiments)<br>IncuCyte ZOOM 2018A (Sartorius; for T cell apoptosis assay using the Incucyte system)<br>SymPhoTime 64 version 2.6 (PicoQuant; for fluorescence-lifetime imaging microscopy)<br>Zen 3.4 (Zeiss; for immunofluorescence stainings)<br>ChromNAV version 1.0 (Jasco; for fast performance liquid chromatography)<br>Amersham Imager 600 with integrated analysis software (GE Healthcare; for developing Western blots)
Data analysis	FlowJo version 10.6.2 (FlowJo; for flow cytometry data)<br>GraphPad Prism version 9 (GraphPad Software; for statistical analyses and generation of graphs)<br>ImageJ 1.51k_Java 1.6.0_24 (NIH; for immunofluorescence, histological, and Western blot data analysis)<br>FLIMfit 5.1.1 (FLIMfit, Open Microscopy Environment; for fluorescence-lifetime imaging microscopy data analysis)

For manuscripts utilizing custom algorithms or software that are central to the research but not yet described in published literature, software must be made available to editors and reviewers. We strongly encourage code deposition in a community repository (e.g. GitHub). See the Nature Portfolio guidelines for submitting code & software for further information.

Data

Policy information about availability of data

All manuscripts must include a data availability statement. This statement should provide the following information, where applicable:

- Accession codes, unique identifiers, or web links for publicly available datasets
- A description of any restrictions on data availability
- For clinical datasets or third party data, please ensure that the statement adheres to our policy

The authors declare that the data supporting the findings of this study are available within the paper and its supplementary information files. All the raw data generated in this study are provided in the Source Data file. Source data are provided with this paper.

Field-specific reporting

Please select the one below that is the best fit for your research. If you are not sure, read the appropriate sections before making your selection.

☒ Life sciences ☐ Behavioural & social sciences ☐ Ecological, evolutionary & environmental sciences

Life sciences study design

All studies must disclose on these points even when the disclosure is negative.

Sample size	Sample size was determined based on extensive experience with similar experiments in our laboratory (Westerterp et al., Circ Res 2013, PMID: 23572498; Westerterp et al., Cell Metabolism 2017, PMID: 28479366). Exact sample size for each experiment is indicated in the figure legends for each panel.
Data exclusions	Data were excluded in two conditions. First, individual data points were excluded if they were identified as outliers by the ROUT test (Q=1%) using GraphPad Prism (version 9). Second, data were excluded when a sample failed due to technical issues. For example, in atherosclerosis studies, when sections were missing or damaged (i.e. one or more aortic valves were broken or missing), the sample was deemed not useable because it would otherwise lead to an unreliable measurement. All exclusions are listed in the Source Data file.
Replication	All attempts of replication were successful. Key experiments were performed at least twice. Exact number of biological replicates can be found in the figure legends.
Randomization	All mice/samples were analysed and allocated randomly.
Blinding	All experiments were performed in a blinded fashion. Mice were assigned unique experimental numbers. After data collection and analysis, these unique numbers were traced back to original mouse numbers in order to perform statistical analysis.<br><br>Also, image acquisition and analysis (apoptosis assay using the Incucyte system, atherosclerotic lesion area (including necrotic core area), CD3 staining in atherosclerotic lesions, Mac-2 and TUNEL+Mac-2 stainings, α-SMA staining, Sirius Red staining, Oil Red O staining, electron microscopy, and fluorescence-lifetime imaging microscopy) were performed in a blinded fashion, i.e. the observer was unaware of the genotypes.

Reporting for specific materials, systems and methods

We require information from authors about some types of materials, experimental systems and methods used in many studies. Here, indicate whether each material, system or method listed is relevant to your study. If you are not sure if a list item applies to your research, read the appropriate section before selecting a response.

Materials & experimental systems

n/a	Involved in the study	
☐	☒	Antibodies
☒	☐	Eukaryotic cell lines
☒	☐	Palaeontology and archaeology
☐	☒	Animals and other organisms
☒	☐	Human research participants
☒	☐	Clinical data
☒	☐	Dual use research of concern

Methods

n/a	Involved in the study	
☒	☐	ChIP-seq
☐	☒	Flow cytometry
☒	☐	MRI-based neuroimaging

Antibodies

Antibodies used

Rat anti-mouse CD115-PE; Biolegend; Cat# 135506; RRID:AB_1937253; clone AFS98; 1/200 dilution
Rat anti-mouse Ly6-C/G-PerCP-Cy5.5; BD Biosciences ;Cat# 561103; RRID:AB_10562568; clone RB6-8C5; 1/200 dilution
Rat anti-mouse CD45-APC-Cy7; BD Biosciences; Cat# 557659; RRID:AB_396774; clone 30-F11; 1/200 dilution
Rat anti-mouse CD25-PECy7; eBioscience; Cat# 25-0251-82; RRID:AB_469608; clone PC61.5; 1/200 dilution
Rat anti-mouse CD4-PB; Biolegend; Cat# 100427; RRID:AB_493646; clone GK1.5; 1/200 dilution
Rat anti-mouse CD4-APC; eBioscience; Cat# 17-0041-82; RRID:AB_469320; clone GK1.5; 1/200 dilution
Rat anti-mouse CD4-APC-Cy7; Biolegend; Cat# 100413; RRID:AB_312698; clone GK1.5; 1/200 dilution
Rat anti-mouse CD4-PE; Biolegend; Cat# 100407; RRID:AB_312692; clone GK1.5; 1/200 dilution
Rat anti-mouse CD4-FITC; eBioscience; Cat# 11-0042-82; RRID:AB_464896; clone RM4-5; 1/200 dilution
Rat anti-mouse CD8-PB (eFluor 450); eBioscience; Cat# 48-0083-82; RRID:AB_11218504; clone eBioH35-17.2 (H35-17.2); 1/200 dilution
Rat anti-mouse CD8-FITC; eBioscience; Cat# 11-0083-82; RRID:AB_657764; clone eBioH35-17.2 (H35-17.2); 1/200 dilution
Rat anti-mouse CD8-PE; eBioscience; Cat# 12-0083-82; RRID:AB_657767; clone eBioH35-17.2 (H35-17.2); 1/200 dilution
Rat anti-mouse CD8-APC Biolegend Cat# 126613; RRID:AB_657767; clone YTS156.7.7; 1/200 dilution
Armenian hamster anti-mouse TCRβ-PB; Biolegend; Cat# 109226; RRID:AB_1027649; clone H57-597; 1/200 dilution
Armenian hamster anti-mouse TCRβ-APC; Biolegend; Cat# 109211; RRID:AB_313434; clone H57-597; 1/200 dilution
Armenian hamster anti-mouse TCRβ-PerCP-Cy5.5; Biolegend; Cat# 109227; RRID:AB_1575176; clone H57-597; 1/200 dilution
Armenian hamster anti-mouse PD1-PB (eFluor 450); eBioscience; Cat# 48-9985; RRID:AB_2574138; clone J43; 1/200 dilution
Rat anti-mouse PD1-PE; Biolegend; Cat# 109103; RRID:AB_313420; clone RMP1-30; 1/200 dilution
Mouse anti-mouse Tbet-PE; eBioscience; Cat# 12-5825; RRID:AB_925761; clone eBio4B10 (4B10); 1/100 dilution
Mouse IgG1 kappa isotype control-PE (Tbet- and Eomes-PE); eBioscience; Cat# 12-4714-42; RRID:AB_470060; clone P3.6.2.8.1; 1/100 dilution
Mouse anti-mouse Tbet-AF488; eBioscience; Cat# 53-5825-80; RRID:AB_2815215; clone eBio4B10 (4B10); 1/100 dilution
Mouse IgG1 kappa isotype control-AF488 (Tbet-AF488); eBioscience; Cat# 53-4714-80; RRID:AB_470230; clone P3.6.2.8.1; 1/100 dilution
Rat anti-mouse Eomes-PE; eBioscience ;Cat# 12-4875-82; RRID:AB_1603275; clone Dan11mag; 1/100 dilution
Rat anti-mouse CXCR5-FITC; Biolegend; Cat# 145519; RRID:AB_2562865; clone L138D7; 1/400 dilution
Rat anti-mouse CD44-PB (eFluor 450); eBioscience; Cat# 48-0441-82; RRID:AB_1272246; clone IM7; 1/200 dilution
Rat anti-mouse CD44-PE-Cy7; eBioscience; Cat# 25-0441-82; RRID:AB_469623; clone IM7; 1/200 dilution
Rat anti-mouse CD62L-APC; eBioscience; Cat# 17-0621-82; RRID:AB_469410; clone MEL-14; 1/200 dilution
Rat anti-mouse CD62L-FITC; Biolegend; Cat# 104405; RRID:AB_313092; clone MEL-14; 1/200 dilution
Rat anti-mouse CD62L-PB Biolegend Cat# 104423; RRID:AB_493381; clone MEL-14; 1/200 dilution
Rat anti-mouse Foxp3-APC; eBioscience; Cat# 17-5773-80; RRID:AB_469456; clone FJK-16s; 1/100 dilution
Rat IgG2a kappa isotype control-APC (Foxp3-APC); eBioscience; Cat# 17-4321-81; RRID:AB_470181; clone eBR2a; 1/100 dilution
Mouse anti-mouse Fas-AF488; eBioscience; Cat# 53-0951-82; RRID:AB_10671269; clone 15A7; 1/200 dilution
Mouse anti-mouse Bcl2-FITC; eBioscience; Cat# 11-6992-42; RRID:AB_10734060; clone 10C4; 1/40 dilution
Mouse IgG1 kappa isotype control-FITC (Bcl2-FITC); eBioscience; Cat# 11-4714-82; RRID:AB_470022; 1/40 dilution
Armenian hamster anti-mouse CTLA4-Brilliant Violet 421 (PB); Biolegend; Cat# 106311; RRID:AB_10901170; clone UC10-4B9; 1/200 dilution
Rat anti-mouse TIM3-APC; Biolegend; Cat# 134007; RRID:AB_2562997; clone B8.2C12; 1/200 dilution
Rat anti-mouse LAG3-APC; Biolegend; Cat# 125209; RRID:AB_10639935; clone C9B7W; 1/200 dilution
Rat anti-mouse CD24-PE-Cy7 Biolegend Cat# 101821; RRID:AB_756047; clone M1/69; 1/200 dilution
Rat anti-mouse CD69-APC Biolegend Cat# 104513; RRID:AB_492844; clone H1.2F3; 1/200 dilution
Rat anti-mouse Granzyme B-FITC eBioscience Cat# 11-8898-80; RRID:AB_10732989; clone NGZB; 1/100 dilution
Rat IgG2a kappa Isotype Control-FITC (Granzyme B-FITC) eBioscience Cat# 11-4321-80; RRID:AB_1834375; 1/100 dilution
Rat anti-mouse IFNγ-FITC eBioscience Cat# 11-7311-81; RRID:AB_465411; clone XMG1.2; 1/100 dilution
Rat anti-mouse LAMP-1 (CD107a) Biolegend Cat# 121605; RRID:AB_572006; clone 1D4B; 1/200 dilution

Rabbit anti-mouse SAPK/JNK (for Western blot) Cell Signaling Technology Cat# 9252; RRID:AB_2250373; 1/1000 dilution
Mouse anti-mouse phospho-SAPK/JNK (Thr183/Tyr185) (for Western blot) Cell Signaling Technology Cat# 9255; RRID:AB_2307321; 1/1000 dilution
Rabbit anti-mouse HSP90 (for Western blot) Cell Signaling Technology Cat# 4874; RRID:AB_2121214; 1/1000 dilution
Rat anti-mouse Gasdermin D (Clone 17G2G9; for Western blot; kind gift from Genentech); 1/1000 dilution
Goat anti-Mouse IgG (H+L) Secondary Antibody, HRP (for Western blot; for Phospho-SAPK/JNK) Invitrogen Cat# 31430; RRID:AB_228307; 1/2000 dilution
Goat anti-Rabbit IgG (H+L) Secondary Antibody, HRP (for Western blot; for SAPK/JNK and HSP90) Invitrogen Cat# 31460; RRID:AB_228341; 1/2000 dilution
Goat anti-rat IgG, HRP-linked (for Western blot; for gasdermin D) Cell Signaling Technology Cat# 7077; AB_10694715; 1/1000 dilution

Rabbit anti-mouse Actin, Smooth Muscle (for histology) Lab Vision Cat# Epredia RB-9010-P; RRID:AB_149757; 1/200 dilution
Rabbit anti-human CD3 (for histology) Dako Cat# A0452; RRID:AB_2335677; 1/250 dilution
Goat anti-rabbit IgG antibody (H+L), biotinylated (for SMA and CD3 stainings) Vector Laboratories Cat# BA-1000; AB_2313606; 1/250 dilution
Rat Anti-Mouse/Human Mac-2 (Galectin-3) (for histology) Cedarlane Cat# CL8942AP; RRID:AB_2534074;clone M3/38; 1/10000 dilution
Goat anti-Rat IgG (H+L) Cross-Adsorbed Secondary Antibody, AF488 (for Mac-2 staining) Invitrogen Cat# A-11006; RRID:AB_10060357; 1/200 dilution
Goat anti-rat IgG antibody (H+L), biotinylated (for Mac-2 staining) Vector Laboratories Cat# BA-9400; AB_ 2336202; 1/125 dilution

LEAF Purified rat anti-mouse CD3 (for stimulation); Biolegend; Cat# 100208; RRID:AB_312665; clone 17A2; amount 5μg/mL

For flow cytometry antibodies, Lot numbers cannot reasonably be provided, as multiple different Lots have been used over the

course of this study. Depending on the individual experimental setting, flow cytometry antibodies from the same clone but coupled to different fluorochromes were used.

Validation

The rat anti-mouse gasdermin D antibody was a kind gift from Genentech. The validation of this antibody was previously performed (Aglietti et al., Proc Natl Acad Sci U S A 2016; cited in the manuscript). All other antibodies were obtained from commercial sources, and validation data and detailed information can be found on the manufacturer's website (Biolegend, eBioscience, BD Biosciences, Cell Signaling Technology, Invitrogen, Lab Vision, Dako, Vector Laboratories, Cedarlane).

Animals and other organisms

Policy information about studies involving animals; ARRIVE guidelines recommended for reporting animal research

Laboratory animals

Mouse: Abca1fl/flAbcg1fl/fl: B6.Cg-Abca1tm1Jp Abcg1tm1Tall/J The Jackson Laboratory Cat# JAX:021067; RRID:IMSR_JAX:021067

Mouse: CD4Cre: Tg(Cd4-cre)1Cwi/BfluJ The Jackson Laboratory Cat# JAX:017336; RRID:IMSR_JAX:017336

Mouse: Ldlr-/-: B6.129S7-Ldlrtm1Her/J The Jackson Laboratory Cat# JAX:002207; RRID:IMSR_JAX:002207

Mouse: wild-type: C57BL/6J The Jackson Laboratory Cat# JAX:000664; RRID:IMSR_JAX:000664

For all experiments age and sex matched littermate controls were used. For atherosclerosis studies, female mice were used. For all other studies, male and female mice were used. Mice were 3-24 months of age, depending on the respective experiments (age specified in figure legends).

Wild animals

No wild animals were used in this study.

Field-collected samples

No field collected samples were used in this study.

Ethics oversight

All protocols were approved by the Institutional Animal Care and Use Committee from the University of Groningen (Groningen, the Netherlands) under permit number AVD105002015244 and adhered to guidelines set out in the 2010/63/EU directive.

Note that full information on the approval of the study protocol must also be provided in the manuscript.

Flow Cytometry

Plots

Confirm that:

☒ The axis labels state the marker and fluorochrome used (e.g. CD4-FITC).

☒ The axis scales are clearly visible. Include numbers along axes only for bottom left plot of group (a 'group' is an analysis of identical markers).

☒ All plots are contour plots with outliers or pseudocolor plots.

☒ A numerical value for number of cells or percentage (with statistics) is provided.

Methodology

Sample preparation

Briefly, blood, spleens, para-aortic lymph nodes (LN), and thymi were collected from mice for flow cytometry analyses. Spleens, LNs, and thymi were mashed. Red blood cells were lysed. White blood cells and whole splenic, LN, and thymic homogenates were stained for surface markers of interest using fluorescent antibodies (30min; on ice; in the dark). For intracellular stainings, after surface marker staining, cells were fixed, permeabilized, and stained for intracellular markers of interest using fluorescent antibodies (30min; on ice; in the dark). Detailed protocols are included in the methods section of this manuscript.

Instrument

BD LSR II flow cytometer

Software

FACS DiVa software V8.0.3 (BD Bioscience; for acquisition of flow cytometry data
FlowJo version 10.6.2 (FlowJo; for flow cytometry data)
GraphPad Prism version 9 (GraphPad Software; for statistical analyses and generation of graphs)

Cell population abundance

No cell sorting was performed in this study

Gating strategy

General gating strategy for T cell analyses:
FSC-A v. SSC-A (cell gate excluding debris and dead cells) --> FSC-A v. FSC-H (doublet exclusion) --> TCRβ v. SSC-A (gating on TCRβ+ cells to select T cells) --> CD8 v. CD4 (gating on CD8+CD4- and CD8-CD4+ populations) --> For further T cell subsets, cells were stained for surface and intracellular markers of interest using additional fluorescent antibodies (detailed information about the stainings are included in the methods section of this manuscript).

For T cell proliferation, cells were labeled with CFSE and the dilution of the dye was assessed.
To assess expression of the markers filipin, choleratoxin B, cleaved caspase3/7, LAMP-1, TIM3, LAG3, CTLA4, Eomes, FAS, MitoSOX, and Mitotracker green, the mean fluorescence intensity (mfi) of these markers in T cells was assessed.

Gating strategy for myeloid cells:
FSC-A v. SSC-A (cell gate excluding debris and dead cells) --> FSC-A v. FSC-H (doublet exclusion) --> CD45 v. SSC-A (gating on CD45+ cells to select leukocytes) --> CD115 v. Ly6C/G --> (gating on CD115+Ly6C/G- to select monocytes; gating on CD115-Ly6C/G+ to select neutrophils) --> CD115 v. Ly6C/G (gating on CD115+Ly6C/G- and CD115+Ly6C/G+ to select Ly6Clo and Ly6Chi monocytes subsets).

As a rule of thumb, boundaries between positive and negative populations were set at approximately 10^3. For most stainings, Fluorescence Minus One (FMO) controls were included to set the gating properly. For intracellular stainings, positive populations were gated based on and corrected for their respective isotype control or the FMO control.

☒ Tick this box to confirm that a figure exemplifying the gating strategy is provided in the Supplementary Information.

This checklist template is licensed under a Creative Commons Attribution 4.0 International License, which permits use, sharing, adaptation, distribution and reproduction in any medium or format, as long as you give appropriate credit to the original author(s) and the source, provide a link to the Creative Commons license, and indicate if changes were made. The images or other third party material in this article are included in the article's Creative Commons license, unless indicated otherwise in a credit line to the material. If material is not included in the article's Creative Commons license and your intended use is not permitted by statutory regulation or exceeds the permitted use, you will need to obtain permission directly from the copyright holder. To view a copy of this license, visit http://creativecommons.org/licenses/by/4.0/
